# Geographical Distribution of Adolescent Body Height with Respect to Effective Day Length in Japan: An Ecological Analysis

**DOI:** 10.1371/journal.pone.0050994

**Published:** 2012-12-05

**Authors:** Masana Yokoya, Hideyasu Shimizu, Yukito Higuchi

**Affiliations:** 1 Shimonoseki Junior College, 1-1 Sakurayama-cho, Shimonoseki, Yamaguchi, Japan; 2 Toshiwa-kai Hospital, 5-8-1 Kanayama, Naka-ku, Nagoya, Japan; 3 Kyushu Kyoritsu University, 1-8 Jiyugaoka, Yahatanishi-ku, Kitakyushu, Japan; Chancellor College, University of Malawi, Malawi

## Abstract

The height of Japanese youth raised in the northern region tends to be greater than that of youth raised in the southern region; therefore, a geographical gradient in youth body height exists. Although this gradient has existed for about 100 years, the reasons for it remain unclear. Consideration of the nutritional improvement, economic growth, and intense migration that has occurred in this period indicates that it is probably the result of environmental rather than nutritional or genetic factors. To identify possible environmental factors, ecological analysis of prefecture-level data on the body size of 8- to 17-year-old youth averaged over a 13-year period (1996 to 2008) and Japanese mesh climatic data on the climatic variables of temperature, solar radiation, and effective day length (duration of photoperiod exceeding the threshold of light intensity) was performed. The geographical distribution of the standardized height of Japanese adolescents was found to be inversely correlated to a great extent with the distribution of effective day length at a light intensity greater than 4000 lx. The results of multiple regression analysis of effective day length, temperature, and weight (as an index of food intake) indicated that a combination of effective day length and weight was statistically significant as predictors of height in early adolescence; however, only effective day length was statistically significant as a predictor of height in late adolescence. Day length may affect height by affecting the secretion of melatonin, a hormone that inhibits sexual and skeletal maturation, which in turn induces increases in height. By affecting melatonin production, regional differences in the duration of the photoperiod may lead to regional differences in height. Exposure to light intensity greater than 4000 lx appears to be the threshold at which light intensity begins to affect the melatonin secretion of humans who spend much of their time indoors.

## Introduction

The results of several ecological analyses of nationwide data indicate that the body height of Japanese youth raised in Northern Japan tends to be greater than that of youth raised in Southern Japan, indicating the existence of a geographical gradient in youth height [1]–[3]. On the basis of his analysis of Japanese adults approximately 20 years of age, who had been born between 1916 and 1927, Kouchi [2] reported a negative geographical correlation between height and average temperature. Likewise, on the basis of their analysis of prefectural data collected between 1958 and 1993, Endo et al. [3] reported a negative correlation between the body size of 17-year-old Japanese adolescents and the annual mean temperature of prefectural capitals, as well as the existence of a geographical (latitudinal) gradient in adolescent body size. Although this gradient has existed for about 100 years, the reasons for it remain unclear.

Such reports of a correlation between physique and temperature indicate that the anatomy of humans living in colder climates tends to conform to Bergmann’s rule, which posits that humans raised in colder climates tend to have larger body sizes than those raised in warmer climates [4]. However, the physiological mechanism underlying this rule remains unclear, like that regarding the gradient in adolescent body size, and some reports have indicated inconsistency in its manifestation [5]–[7].

Rapid post-World War II economic growth and improvements in nutrition have resulted in great development in the physique of Japanese youth [8], with the generally very poor physique of Japanese youth having gradually improved since 1945. The Japanese Ministry of Education, Culture, Sports, Science and Technology reported that the average maximum development of the physique of Japanese youth peaked between1997 and 2001, and has since remained at this high level [8]. Despite such improvement, regional differences in physique and a geographical gradient in physique continue to exist. Considering that this gradient is known to have existed for approximately 100 years and that efforts for nutritional improvement have existed for approximately 100 years, a period over which economic growth and rapid migration of rural populations to urban areas occurred, this gradient cannot be attributed to nutritional, socioeconomic, or genetic factors but probably to natural environmental factors.

Among these factors, climatic environment may be a cause of the geographical gradient because it has not changed over the past 100 years. In particular, the photoperiodic environment has recently been attracting attention from various fields, as it affects the physiological processes of all living organisms and initiates many biological and behavioral changes. Many studies have shown that the photoperiodic environment can have many different effects on behavior, growth, reproduction, and some diseases [9]–[11].

To clarify the influence of possible environmental factors, it is necessary to study in detail the relationship between geographical differences in body size and climatic and photoperiodic factors, which have not been investigated previously. To fulfill this research need, we conducted ecological analysis of prefecture-level data regarding the anatomical variables (height and weight) of Japanese youth; temperature; and the climatic factors closely related to temperature, including solar radiation, sunshine duration, and effective day length (the duration of photoperiod exceeding the threshold of light intensity). Since climatic factors generally correlate with each other, it was necessary to distinguish the influence of each climatic factor on anatomical factors. For this reason, detailed analysis was performed using data obtained from the geographical information system (GIS) and mesh climatic data [12]. GIS is a system designed to capture, store, manipulate, analyze, and present all types of geographical data. By using GIS, we could perform more accurate spatial analysis. This analysis resulted in findings of strong geographical correlation between Japanese adolescent body height and climatic factors other than temperature and the development of a hypothesis regarding the most significant factor in the height gradient that exists among Japanese adolescents.

## Results

Table 1 shows the basic statistics regarding the height and weight of Japanese youth standardized over a 13-year period from 1996 to 2008. As can be observed, the maximum heights were observed in northern prefectures (Akita, Aomori, and Niigata), and the minimum heights were observed in southern prefectures (Okinawa, Miyazaki, and Yamagichi). Standard deviation in the standardized height of 14-year-old males and females raised in each prefecture over the 13-year-period was found to be 0.02 to 0.06 (approximately 0.1 to 0.5 cm for a 14-year-old male and 0.1 to 0.3 cm for a 14-year-old of female; see Table S1). The geographical difference between the 2 extreme values in body height (approximately 2.4 to 3.2 cm for a 14-year-old male and 2.1 to 3.2 cm for a 14-year-old female) was found to be much larger than the annual fluctuation in body height in each prefecture. Similarly, the maximum weights were observed in northern prefectures (Aomori and Iwate), and the minimum weights were observed in southern prefectures (Shimane, Okinawa, and Yamaguchi). Standard deviation in the standardized weight of 14-year-old males and females raised in these prefectures over the 13-year-period was found to be 0.02 to 0.06 (approximately 0.3 to 0.8 kg for a 14-year-old male and 0.2 to 0.6 kg for a 14-year-old female; see Table S2). The geographical difference between the 2 extreme values in body weight (approximately 3.9 to 5.4 kg for a 14-year-old male and 2.6 to 3.5 kg for a 14-year-old female) was found to be much larger than the annual fluctuation in the respective body weight for each prefecture.

**Table 1:** Basic statistics for standardized height and weight.

Standardized height	Males			Females		
Age	10	14	17	10	14	17
Maximum	0.263(Akita)	0.221(Akita)	0.169(Akita)	0.254(Aomori)	0.171(Akita)	0.135(Niigata)
Minimum	−0.217(Okinawa)	−0.165(Miyazaki)	−0.302(Okinawa)	−0.119(Yamaguchi)	−0.305(Okinawa)	−0.311(Okinawa)
Mean	−0.002	−0.007	−0.009	0.006	−0.013	−0.011
Median	−0.009	−0.022	−0.004	−0.016	−0.023	−0.002
**Standardized weight**	**Males**			**Females**		
**Age**	**10**	**14**	**17**	**10**	**14**	**17**
Maximum	0.289(Aomori)	0.258(Aomori)	0.240(Aomori)	0.314(Aomori)	0.231(Aomori)	0.197(Iwate)
Minimum	−0.150(Shimane)	−0.152(Yamaguchi)	−0.142(Yamaguchi)	−0.100(Yamaguchi)	−0.105(Okinawa)	−0.238(Okinawa)
Mean	0.006	−0.001	0.002	0.016	0.010	0.012
Median	−0.028	−0.012	−0.011	−0.001	−0.013	0.002

Table 2 shows the results of analysis of 30-year average population-weighted climatic data in each prefecture. The maximum annual mean temperature, annual mean solar radiation, annual total sunshine duration, and annual mean effective day length were observed in southern prefectures (Okinawa and Kochi), and the minimum values were observed in northern prefectures (Hokkaido, Niigata, and Akita). According to analysis of the data collected at 41 prefectural-capital weather stations between 1971 and 2000 [13], the standard deviation of the annual mean temperature at these sites over the 30-year-period was 0.52 to 0.89°C, and the standard deviation of the annual mean global solar radiation was 0.43 to 1.10 MJ/m^2^/day. The geographical difference between the 2 extreme values of these climatic variables was found to be much larger than the annual fluctuation in the respective climatic variables for each prefectural-capital weather station (see Table S3), with the geographical difference between the 2 extreme values in terms of annual mean effective day length at 4000 lx found to be 0.38 h/day.

**Table 2:** Basic statistics for climatic variables.

	Annual mean temperature (°C)	Annual mean solar radiation (MJ/day)	Annual total sunshine duration (h)	Annual meaneffective daylength at 1000 lx(h/day)	Annual meaneffective daylength at 4000 lx(h/day)	Annual meaneffective daylength at 10000 lx(h/day)
Maximum	22.3(Okinawa)	14.00(Kochi)	2104(Kochi)	11.70(Kochi)	10.80(Kochi)	9.03(Kochi)
Minimum	7.1(Hokkaido)	11.70(Niigata)	1570(Niigata)	11.60(Niigata)	10.42(Akita)	8.05(Akita)
Mean	14.1	12.78	1861	11.65	10.63	8.57
Median	14.6	12.80	1883	11.65	10.63	8.60

Table 3 shows the correlation matrix of climatic variables as determined by Pearson’s correlation coefficient analysis of the set of population-weighted climatic values obtained in the 47 prefectures. The Spearman’s correlation coefficient for each variable was found to be almost the same as the corresponding Pearson’s correlation coefficient. In addition, most of the climatic variables were significantly correlated with each other, and the annual mean global solar radiation and effective day length were correlated in an almost directly proportional manner.

**Table 3:** Pearson correlation matrix of climatic variables.

	TMP	SOLA	SUN	1000	2000	3000	4000	5000	10000
TMP	1								
SOLA	0.56**	1							
SUN	0.35	0.76**	1						
1000	0.58**	0.95**	0.80**	1					
2000	0.62**	0.94**	0.82**	0.96**	1				
3000	0.63**	0.95**	0.84**	0.96**	0.97**	1			
4000	0.62**	0.95**	0.85**	0.96**	0.98**	0.99**	1		
5000	0.61**	0.96**	0.86**	0.96**	0.98**	0.99**	1.00**	1	
10000	0.62**	0.96**	0.86**	0.96**	0.98**	0.99**	1.00**	1.00**	1

TMP: Annual mean temperature.

SOLA: Annual mean solar radiation.

SUN: Annual total sunshine duration.

1000–10000: Annual mean effective day length at 1000 to 10000 lx.

** p<0.0001.

* p<0.005.

Table 4 shows the Pearson’s correlation coefficients of the relationship between the height of 8- to 17-year-old youth standardized over the 13-year study period and 30-year average values of several climatic variables. The Spearman’s correlation coefficient for each variable was found to be almost the same as the corresponding Pearson’s correlation coefficient. The correlation coefficient between height and weight was added as an index of food intake on the assumption that the extent to which equilibrium weight is maintained is directly proportional to the level of food intake [14], [15]. Height was found to be inversely correlated to a significant extent with all the climatic variables examined, but was inversely correlated to a greater extent with annual mean global solar radiation and effective day length than with temperature. The correlation coefficients for annual mean global solar radiation were found to be the strongest for 14-year-old males (r = −0.88) and 13-year-old females (r = −0.89), while the correlation coefficients for effective day length were found to be the strongest for 14-year-old males (r = −0.89 at 4000 to 10000 lx) and 12- to 13-year-old females (r = −0.88 at 2000 to 10000 lx). Further analysis indicated that the correlation coefficient between height and annual mean global solar radiation or effective day length at 2000 to 10000 lx was stronger than that between height and weight for males aged ≥13 years and for females aged ≥11 years.

**Table 4:** Pearson’s correlation coefficients of the relationship between height and climatic variables.

	TMP	SOLA	SUN	1000	2000	4000	10000	Weight
**Males**								
Age: 8	−0.76**	−0.80**	−0.56**	−0.76**	−0.81**	−0.81**	−0.81**	0.80**
9	−0.79**	−0.81**	−0.58**	−0.78**	−0.83**	−0.83**	−0.83**	0.84**
10	−0.79**	−0.79**	−0.59**	−0.75**	−0.81**	−0.82**	−0.82**	0.87**
11	−0.72**	−0.76**	−0.59**	−0.72**	−0.79**	−0.80**	−0.80**	0.87**
12	−0.70**	−0.77**	−0.62**	−0.73**	−0.80**	−0.82**	−0.82**	0.86**
13	−0.68**	−0.82**	−0.68**	−0.79**	−0.84**	−0.86**	−0.87**	0.84**
14	−0.66**	−0.88**	−0.68**	−0.84**	−0.88**	−0.89**	−0.89**	0.77**
15	−0.62**	−0.87**	−0.64**	−0.85**	−0.87**	−0.87**	−0.87**	0.66**
16	−0.65**	−0.86**	−0.61**	−0.84**	−0.86**	−0.85**	−0.86**	0.65**
17	−0.62**	−0.85**	−0.62**	−0.84**	−0.85**	−0.85**	−0.85**	0.60**
**Females**								
Age: 8	−0.72**	−0.80**	−0.67**	−0.78**	−0.83**	−0.84**	−0.84**	0.83**
9	−0.69**	−0.76**	−0.68**	−0.75**	−0.80**	−0.82**	−0.82**	0.86**
10	−0.64**	−0.72**	−0.67**	−0.71**	−0.76**	−0.79**	−0.79**	0.86**
11	−0.65**	−0.78**	−0.71**	−0.77**	−0.81**	−0.84**	−0.84**	0.76**
12	−0.67**	−0.85**	−0.70**	−0.82**	−0.87**	−0.88**	−0.88**	0.59**
13	−0.63**	−0.89**	−0.66**	−0.86**	−0.88**	−0.88**	−0.88**	0.54**
14	−0.62**	−0.86**	−0.62**	−0.83**	−0.85**	−0.84**	−0.85**	0.49**
15	−0.61**	−0.83**	−0.56**	−0.79**	−0.81**	−0.80**	−0.81**	0.51**
16	−0.59**	−0.81**	−0.51**	−0.78**	−0.77**	−0.76**	−0.77**	0.54**
17	−0.60**	−0.83**	−0.57**	−0.82**	−0.81**	−0.80**	−0.81**	0.57**

TMP: Annual mean temperature.

SOLA: Annual mean solar radiation.

SUN: Annual total sunshine duration.

1000–10000: Annual mean effective day length at 1000 to 10000 lx.

** p<0.0001

* p<0.005.

Table 5 shows the Pearson’s correlation coefficients for the relationship between the weight of 8- to 17-year-old youth standardized over the 13-year study period and 30-year average values of several climatic variables. The Spearman’s correlation coefficient for each variable was found to be almost the same as the corresponding Pearson’s correlation coefficient. However, whereas weight was found to be strongly inversely correlated with all the climatic variables to an equal extent, increasing with decrease in temperature, solar radiation, or effective day length, weight was found to be inversely correlated to a greater extent with temperature than with annual mean global solar radiation or effective day length. In contrast to those for height, the correlation coefficients for the relationship between weight and the climatic variables for 11- to 14-year-olds were found to be weak.

**Table 5:** Pearson correlation coefficients of the relationship between weight and climatic variables.

	TMP	SOLA	SUN	1000	2000	4000	10000	Height
**Males**								
Age: 8	−0.69**	−0.55**	−0.42**	−0.53**	−0.58**	−0.59**	−0.59**	0.80**
9	−0.71**	−0.56**	−0.42**	−0.54**	−0.59**	−0.61**	−0.60**	0.84**
10	−0.71**	−0.54**	−0.39**	−0.51**	−0.57**	−0.59**	−0.58**	0.87**
11	−0.65**	−0.51**	−0.36*	−0.48**	−0.53**	−0.56**	−0.55**	0.87**
12	−0.64**	−0.47**	−0.32*	−0.44**	−0.50**	−0.52**	−0.52**	0.86**
13	−0.64**	−0.53**	−0.36*	−0.50**	−0.55**	−0.57**	−0.57**	0.84**
14	−0.64**	−0.58**	−0.38**	−0.55**	−0.60**	−0.61**	−0.61**	0.77**
15	−0.65**	−0.59**	−0.39**	−0.57**	−0.60**	−0.61**	−0.61**	0.66**
16	−0.70**	−0.60**	−0.42**	−0.58**	−0.64**	−0.64**	−0.63**	0.65**
17	−0.73**	−0.62**	−0.45**	−0.62**	−0.67**	−0.67**	−0.66**	0.60**
**Females**								
Age: 8	−0.64**	−0.54**	−0.46**	−0.52**	−0.57**	−0.60**	−0.59**	0.83**
9	−0.61**	−0.50**	−0.45**	−0.50**	−0.54**	−0.56**	−0.56**	0.86**
10	−0.55**	−0.45**	−0.41**	−0.44**	−0.48**	−0.51**	−0.50**	0.86**
11	−0.53**	−0.44**	−0.37*	−0.43**	−0.45**	−0.49**	−0.48**	0.76**
12	−0.56**	−0.38**	−0.34*	−0.38**	−0.43**	−0.45**	−0.44**	0.59**
13	−0.64**	−0.50**	−0.39**	−0.48**	−0.53**	−0.55**	−0.54**	0.54**
14	−0.69**	−0.50**	−0.38**	−0.48**	−0.54**	−0.54**	−0.54**	0.49**
15	−0.72**	−0.56**	−0.41**	−0.56**	−0.58**	−0.58**	−0.58**	0.51**
16	−0.74**	−0.58**	−0.43**	−0.59**	−0.60**	−0.60**	−0.60**	0.54**
17	−0.76**	−0.60**	−0.44**	−0.60**	−0.63**	−0.61**	−0.62**	0.57**

TMP: Annual mean temperature.

SOLA: Annual mean solar radiation.

SUN: Annual total sunshine duration.

1000–10000: Annual mean effective day length at 1000 to 10000 lx.

** p<0.0001, *p<0.005.

The results of the analysis indicate that the correlation between effective day length and height tends to become slightly larger as light intensity increases from 1000 to 4000 lx, before plateauing at values greater than 4000 lx. On the basis of this finding, the data for 14-year-old males and females, an age after which peak height velocity has been achieved by Japanese youth [8], were analyzed in accordance with an effective day length at 4000 lx of light intensity (h/day). However, this estimation did not greatly affect the results because the distributions of effective day length at 1000 to 10000 lx were highly correlated with each other (Table 3).

Table 6 shows the partial correlations found between each climatic variable and the standardized height of 14-year-old males and females, after adjusting for single explanatory variables. When either the annual mean solar radiation or effective day length at 4000 lx was eliminated from the analysis, the partial correlation between height and other climatic variables became insignificant. Table 7 shows the partial correlations found between each climatic variable and the standardized weight of 14-year-old males and females, after adjusting for single explanatory variables. When either the annual mean temperature or height was eliminated from the analysis, the partial correlation between weight and almost every other climatic variable became insignificant.

**Table 6:** Partial correlation coefficients of the relationship between height and climatic variables on the basis of sex.

	Partialed-out variable				
*14-year-old males*	TMP	SOLA	SUN	4000	Weight
TMP	–	0.18	−0.36*	0.29	−0.08
SOLA	−0.46**	–	−0.41**	−0.30*	−0.32*
SUN	−0.39**	0.21	–	0.20	−0.27
4000	−0.46**	−0.36*	−0.47**	–	−0.32*
Weight	0.33*	−0.09	0.44**	−0.18	–
***14-year-old females***					
TMP	–	0.16	−0.36*	0.15	−0.34*
SOLA	−0.49**	–	−0.47**	−0.40**	−0.61**
SUN	−0.36*	0.21	–	0.09	−0.42**
4000	−0.46**	−0.36*	−0.49**	–	−0.59**
Weight	0.10	−0.23	0.22	−0.22	–

TMP: Annual mean temperature.

SOLA: Annual mean solar radiation.

SUN: Annual total sunshine duration.

4000: Annual mean effective day length at 4000 lx.

** p<0.01, *p<0.05.

**Table 7:** Partial correlation coefficients of the relationship between weight and climatic variables on the basis of sex.

	Partialed-out variable				
*14-year-old males*	TMP	SOLA	SUN	4000	Height
TMP	–	−0.32*	−0.51**	−0.27	−0.05
SOLA	−0.19	–	−0.38*	−0.35*	−0.07
SUN	−0.10	−0.03	–	−0.03	0.22
4000	−0.20	−0.40**	−0.43**	–	−0.13
Height	0.34*	0.47**	0.56**	0.46**	–
***14-year-old females***					
TMP	–	−0.43**	−0.56**	−0.38*	−0.42**
SOLA	−0.05	–	−0.30	−0.31*	−0.25
SUN	−0.06	−0.09	–	−0.09	−0.10
4000	−0.07	−0.37*	−0.36*	–	−0.29
Height	0.06	0.24	0.29	0.20	–

TMP: Annual mean temperature.

SOLA: Annual mean solar radiation.

SUN: Annual total sunshine duration.

4000: Annual mean effective day length at 4000 lx.

** p<0.01, *p<0.05.

Table 8 shows the results of multiple linear regression analysis performed to predict the height of 8- to 17-year-old Japanese youth. Later models were developed on the basis of the results of multiple linear regression analysis using a stepwise multivariate procedure, which indicated that only a combination of annual mean global solar radiation and weight or a combination of effective day length at 4000 lx and weight could be used as predictors. Effective day length at 4000 lx was found to be significantly and inversely correlated to a greater extent with height in both sexes, and particularly for 13-year-old males and 12-year-old females. While weight was found to be positively correlated to a significant extent with height for males ≤14 years and for females ≤11 years, the correlation tended to become weaker with age. On the basis of these findings, weight was selected as a primary predictor for males aged ≤12 years and females aged ≤10 years, whereas effective day length at 4000 lx was selected as a primary predictor for males aged ≥13 years and females aged ≥11 years. Further analysis indicated that the addition of any other factor to the model would have no effect and that the percentage of inter-country variability (R^2^) explained by the model decreases with age for males aged ≥12 years and females aged ≥10 years.

**Table 8:** Regression coefficients (standard errors) of predictors of height.

Age of Males		Regression coefficient	Standard error	t	p	R^2^
	4000	−0.51	0.08	−6.33	<0.0001	
8	Weight	0.50	0.08	6.25	<0.0001	0.807
	4000	−0.50	0.07	−7.20	<0.0001	
9	Weight	0.54	0.07	7.71	<0.0001	0.860
	4000	−0.47	0.06	−7.77	<0.0001	
10	Weight	0.59	0.06	9.80	<0.0001	0.891
	4000	−0.46	0.06	−8.24	<0.0001	
11	Weight	0.62	0.06	11.18	<0.0001	0.902
	4000	−0.50	0.05	−10.61	<0.0001	
12	Weight	0.60	0.05	12.70	<0.0001	0.925
	4000	−0.57	0.05	−11.22	<0.0001	
13	Weight	0.52	0.05	10.23	<0.0001	0.921
	4000	−0.68	0.07	−10.02	<0.0001	
14	Weight	0.35	0.07	5.26	<0.0001	0.868
	4000	−0.75	0.09	−8.53	<0.0001	
15	Weight	0.20	0.09	2.31	0.0255	0.779
	4000	−0.74	0.10	−7.50	<0.0001	
16	Weight	0.18	0.10	1.79	0.0802	0.735
	4000	−0.80	0.11	−7.43	<0.0001	
17	Weight	0.07	0.11	0.66	0.5155	0.706
**Age of Females**		**Regression coefficient**	**Standard error**	**t**	**p**	**R^2^**
	4000	−0.53	0.07	−8.11	<0.0001	
8	Weight	0.52	0.07	7.85	<0.0001	0.872
	4000	−0.49	0.06	−8.50	<0.0001	
9	Weight	0.58	0.06	10.15	<0.0001	0.896
	4000	−0.47	0.05	−8.75	<0.0001	
10	Weight	0.62	0.05	11.70	<0.0001	0.903
	4000	−0.61	0.06	−9.61	<0.0001	
11	Weight	0.46	0.06	7.20	<0.0001	0.857
	4000	−0.77	0.07	−10.92	<0.0001	
12	Weight	0.25	0.07	3.47	0.0012	0.816
	4000	−0.82	0.09	−9.63	<0.0001	
13	Weight	0.09	0.09	1.10	0.2756	0.763
	4000	−0.82	0.10	−8.50	<0.0001	
14	Weight	0.05	0.10	0.53	0.6019	0.700
	4000	−0.76	0.11	−6.89	<0.0001	
15	Weight	0.08	0.11	0.69	0.4941	0.629
	4000	−0.68	0.12	−5.70	<0.0001	
16	Weight	0.13	0.12	1.09	0.2833	0.574
	4000	−0.71	0.11	−6.28	<0.0001	
17	Weight	0.13	0.11	1.17	0.2491	0.628

4000: Annual mean effective day length at 4000 lx (h).

## Discussion

Ecological analysis of nationwide data has indicated that the body height of Japanese youth tended to be greater in Northern Japan for at least the past 100 years, indicating the existence of a geographical gradient in youth height over this period [1]–[3]. Although the physique of Japanese youth was very poor at the end of World War II [8], rapid economic growth and strenuous efforts to improve nutritional status have led to great improvements in the physical development of Japanese youth since 1945. The Ministry of Education, Culture, Sports, Science, and Technology reported that after peaking between 1997 and 2001, the physical development of the average youth has remained at a level much higher than that observed in the history of Japan [8]. Nevertheless, a geographical gradient in body height is still observed. Consideration of nutritional improvement and economic growth that have occurred over this period indicates that the gradient is unlikely to be the result of nutritional or socioeconomic factors because these factors have changed greatly over the past 100 years.

Recent societal and lifestyle changes have led to the adoption of more healthful eating habits among the Japanese population. The results of the National Nutritional Survey, which is conducted on an annual basis in Japan, indicate that many Japanese seek to follow a healthy diet. In modern Japan, large-scale malnutrition attributable to socioeconomic reasons does not exist, nor do serious nutritional deficiencies that might affect growth. Since the results also indicated that geographical differences in nutritional intake are small [16]–[18], the geographical gradient in body size cannot be attributed to regional differences in nutritional intake or eating habits. Indeed, no major geographical difference in nutritional intake can be used to explain the nationwide geographical gradient in body size [16]–[18].

The results of the correlation analysis indicated that the correlation between height and global solar radiation and between height and effective day length at 4000 lx were stronger than the correlation between height and weight for males ≥13 years and females aged ≥11 years. On the basis of these findings and their confirmation by multiple regression analysis, weight was not selected as a primary factor to explain the geographical distribution of height among Japanese adolescents. Assuming that weight is an index of food intake (based on the assumption that the extent to which equilibrium weight is maintained is directly proportional to the level of food intake [14], [15]), even analysis of food intake data will not provide results that can explain the geographical gradient in height better than effective day length. Together, these results indicate that differences in nutritional intake cannot account for the geographical gradient in the body height of Japanese adolescents.

Likewise, it appears unlikely that genetic factors can explain the geographical gradient in body height in Japan. Although the gradient could be explained by genetic differences if only a few genes were involved in height determination, hundreds of gene clusters are thought to be related to human height in a complex manner [19], making it difficult to attribute height variation to differences in the expression or distribution of hundreds of genes across the Japanese archipelago. This difficulty is compounded by the fact that intense migrations of populations from rural to urban regions continue to occur across the archipelago [20], which led the populations of urban prefectures to increase 3-fold between 1920 and 2000. Among them, the populations of Kanagawa, Saitama, Chiba, Tokyo, Osaka, and Aichi Prefectures have increased by 6.6-, 5.3-, 4.5-, 3.4-, 3.4-, and 3.5-fold, respectively, over this period [20]. Although these intense population migrations must have led to the mingling of hundreds of height-related genes, the geographical gradient in height is unaffected, indicating that genetic factors cannot explain the geographical gradient in Japanese body height. Indeed, a number of migration flows are occurring toward the central area of Japan. Even in this case, population migrations must have led to the mingling of hundreds of height-related genes. This fact will result in a break in the continuous geographical gradient and not reinforce the geographical gradient.

As summarized by Case and Paxson [21], in Western countries, about 80% of height variation is genetic and about 20% is related to environmental factors. Perhaps the variation in height within each prefecture group is mainly caused by genetic factors. The standard deviation values indicate that the degree of variation within the group is almost the same as within the whole country and each prefecture [8]. Even though the degree of variation within each prefecture is much larger than the differences between the average values of the prefectures, the order of the average values of each prefecture has remained fixed over a long period. This trend indicates that non-genetic factors control the order of the average heights among the prefectures. In relation to this, Steckel [22] shows that genes are important determinants of height in individuals, but genetic differences typically cancel out in comparisons of average height levels between 2 different populations.

According to Bergmann’s rule, the body size of mammals increases proportionally with decreases in climatic temperature because greater body size is advantageous in colder environments [5]–[7]. However, we study found that height is negatively correlated to a greater extent with annual mean solar radiation and effective day length than with temperature. Even more significantly, the results of multiple regression analysis did not lead to selection of temperature as a significant factor in explaining the geographical distribution of height, indicating that temperature is not a primary factor in the geographical gradient in height.

Although height has been negatively correlated with annual mean global solar radiation, no study has found that solar radiation affects bone extension, suggesting that solar radiation does not directly affect body height. While past research has found a positive relationship among solar radiation, vitamin D generation, and bone formation [23]–[25], this study found a negative correlation between height and solar radiation, suggesting an inverse relationship between solar radiation and vitamin D generation. Additionally, as modern humans spend much of the day indoors, it is unlikely that ambient solar radiation or temperature has a direct impact on any biological processes. Moreover, as variations in the amount of solar radiation exposure at the individual level are primarily the result of lifestyle differences rather than geographical differences [26], [27], it is doubtful that a strongly negative relationship could be found between height and solar radiation at the individual level.

Meanwhile, a number of recent studies have provided support for a link between seasonal changes in photoperiodic environment and physiological alterations. It is well known that a circadian clock is involved in the regulation of photoperiodism [28]–[31]. It is hypothesized that in humans, daily variation in the production of melatonin, a hormone that regulates the sleep–wake cycle by chemically inducing drowsiness [28]–[31], serves as a type of seasonal clock and that modulation of circadian rhythms by light is primarily mediated by melanopsin-containing retinal ganglion cells. Melanopsin cells are intrinsically blue-light sensitive, as demonstrated by the inability of an organism to produce melatonin when blue-light levels exceed a certain threshold [32]–[34]. Since daylight includes bright blue light relative to the luminance of room light, all organisms are greatly influenced by the length of the photoperiod.

Melatonin production has also been found to increase the secretion of gonadotropin-inhibitory hormone, which in turn inhibits the secretion of gonadotropin-releasing hormone, but inhibit the secretion of luteinizing hormone and follicle-stimulating hormone from the anterior pituitary gland [35]–[39], which in turn suppresses libido and sexual maturation. Indeed, melatonin production has been found to inhibit sexual maturation [35]–[42] in mammals (including humans), which may delay skeletal maturation and induce height extension [43]. The results of both the correlation analysis and multiple regression analysis performed in this study support these findings, as they indicate the existence of a strongly negative correlation between height and effective day length, particularly among 13-year-old males and 12-year-old females (ages after which Japanese youth attain peak height velocity, which occurs at 11 years for males and 9 to 10 years for females) [8]. These findings indicate the existence of a strongly positive relationship between effective day length and sexual maturation.

Several studies have reported a geographical gradient of age at menarche [44]–[46]. The world distribution of age at menarche shows a decreasing trend as the geographical latitude approaches approximately 25–30 degrees and then increases again toward 0 degrees (near the equator) [44]. The findings of these studies suggest that the amount and quality of sunlight may play a major role in the initiation of menses and sexual maturation. That is, to delay menses and sexual maturation relatively strong light intensity is required, the distribution of which is strongest between 25–30 degrees of latitude; the light intensity is required in the high latitudes because of lower sun elevation and high cloud cover, and less light intensity is required near the equator because of high cloud cover. These distributions can be explained using the concept of the effective day length, because the distribution of effective day length at a light intensity of more than 1000 lx is almost proportional to the distribution of the amount of solar radiation (Table 3).

The study findings also indicated that the correlation between effective day length and height tended to become slightly more negative as light intensity increased from 1000 to 4000 lx. Previous studies have found that a light intensity of 50 to 600 lx can induce considerable phase shifts in the human melatonin–circadian rhythm [47], [48] and that phototherapy for treating sleep–wake rhythm disorders is only effective at a light intensity greater than 1000 lx [49], indicating that a light intensity of 4000 lx is too strong to affect the secretion of melatonin. However, when the modern human lifestyle is taken into consideration–one in which a considerable amount of time is spent indoors–an outdoor light intensity of 4000 lx appears to be an appropriate threshold at which light intensity could begin to affect the melatonin secretion of humans who spend most of their time indoors. In fact, seasonal changes in day length induce seasonal variation in melatonin secretion [50] and physiological changes in the duration of sleep, so that our waking time is earlier in summer and later in winter [51]. This phenomenon is caused by relatively strong daylight that is bright enough to wake individuals who spend most of their time indoors.

In general, an ambient light intensity of 4000 lx is the intensity observed on a cloudy morning and in the evening. Previous research has established that the light intensity of indoor environments is generally less than 10% of that outdoors and that annual total possible sunshine duration is constant across geographical areas [52], [53]. When these facts are considered together with a finding of this study–that the geographical difference between the 2 extreme values in annual mean effective day length at 1000 lx is only 0.10 h/day (Table 2), 2 hypotheses can be proposed: (1) geographical differences in effective day length become smaller with decreases in light intensity (see Formula 7) and (2) geographical differences in effective day length at low light intensities (<1000 lx) are too small to produce a geographical gradient in body height. When these facts are considered in turn with the finding that exposure to illumination varies greatly at the individual level because of geographical rather than lifestyle differences [52], [53], they support the possibility that regional differences in day length are the primary factors in regional differences in height.

Generally, body weight is said to have a more primary association with climatic variables than height [54]; however, the findings of this study do not support this indication. This discrepancy in findings can be explained on the basis of regional differences in sexual maturation. In both males and females, peak weight velocity occurs after attainment of peak height velocity [8]. However, whereas weight gain in adolescent males is largely the result of increases in height and muscle mass, that in adolescent females is largely the result of increases in height and fat mass rather than muscle mass [55]. Since climatic variables, such as temperature, have been found to have a great effect on the basal metabolic rate, weight has been posited as more significantly associated with climatic variables than height [56]. However, adolescents typically experience a “growth spurt” during which weight gain occurs secondary to height gain, a phenomenon that may be responsible for the relatively weak correlation found between weight and effective day length in this study.

Except in Japan, little research has been conducted on the geographical correlation between climate and human body size [54], [57] that may reflect the belief that only nutritional and/or genetic factors are related to body size. Compared to those of most other countries, the Japanese population exhibits few geographical differences in nutritional intake [16]–[18], [58] and is relatively ethnically homogeneous, appearing to have less genetic variation than populations of other countries [59]. This nutritional and genetic uniformity may be responsible for the ability to identify the effects of climate on body height and the existence of a geographical gradient in body height in Japan. Several studies have found that the sensitivity of melatonin to light suppression is influenced by eye pigmentation, which is related to ethnicity [60], as well as the existence of racial differences in the human photoperiod [61], [62]. These findings indicate that the effect of racial/ethnic differences on anatomical factors should be further investigated, and because of the homogeneity of its population, Japan is a particularly apt region in which to do so.

All ecological studies are potentially prone to the ecological fallacy, and our study findings should thus be interpreted cautiously. Although we checked for potential confounders, we cannot exclude that unknown or unmeasured factors may have influenced the study results. Since many studies, including this study, have been conducted using only nationwide published data, additional studies using individual-level data to evaluate the impact of climatic factors on anatomical factors should be performed. Since the impact of environmental factors on the human body varies according to individual lifestyle, the use of group-level data has limited utility when evaluating environmental factors that affect health. In this study, we found that the annual fluctuation in effective day length is relatively large, but we were unable to identify a clear relationship between height and effective day length by using averaged values for body height and climatic variables. In light of these findings, the impact of effective day length may be found to be small at the individual level, as it appears that detecting the influence of effective day length without using group-level and averaged data is difficult. In addition, the physiological mechanism responsible for the regional gradient has not been definitively identified, and it remains unclear whether a regional gradient in sexual maturation that reflects the regional gradient in height exists. Further research is necessary to definitively establish the association between climatic factors and health/anatomical factors.

### Conclusions

To investigate the factors responsible for the geographical gradient in the body height of Japanese youth, ecological analysis was performed using prefecture-level data regarding the height of 8- to 17-year-old Japanese youth that had been averaged over a 13-year period and Japanese mesh climatic data regarding the climatic variables of temperature, solar radiation, and effective day length. The geographical distribution of the standardized height of Japanese youth was found to be closely related to the distribution of effective day length at light intensities greater than 4000 lx, increasing as effective day length decreased. The results of multiple regression analysis indicated that effective day length but not weight (added as an index of food intake) was a statistically significant primary predictor for the geographical gradient in body height that exists among 13- to 17-year-old males and 11- to 17-year-old females.

Since melatonin secretion is affected by day length, differences in melatonin secretion because of variations in day length may be significant factors in the geographical gradient in height. Specifically, by inhibiting sexual maturation and skeletal maturation, melatonin secretion may induce increases in height, and as melatonin secretion is affected by the photoperiod, regional differences in the duration of the photoperiod may lead to regional differences in sexual maturation and height. The results of this study indicate that a light intensity of 4000 lx is the threshold at which light exposure begins to affect the melatonin secretion of humans who lead a typical “indoor” lifestyle. Consequently, geographical differences in effective day length at a light intensity greater than 4000 lx is hypothesized to be the factor that best explains geographical differences in the body height of Japanese youth.

## Materials and Methods

### Ethics Statement

Our study did not require an ethics committee approval because we relied entirely on previously published data.

### Study Area

**Figure 1:**
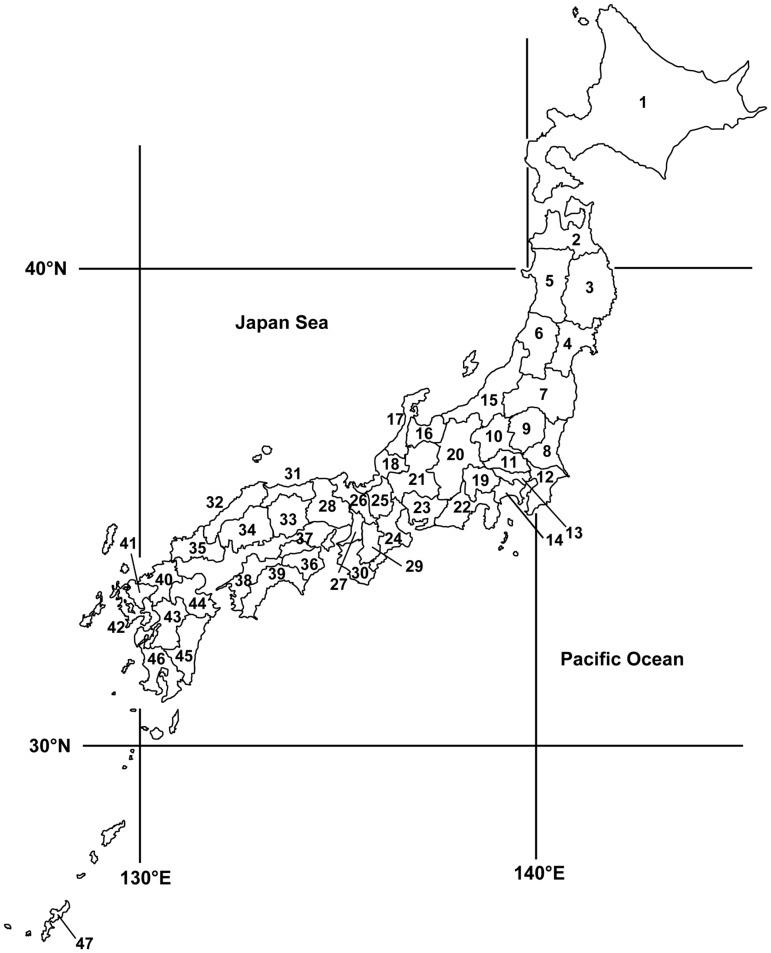
Map of the 47 prefectures of Japan. The numbers correspond to the prefecture information presented in Tables S1, S2, S3.

Figure 1 shows a map of the prefectures in Japan. Japan consists of 47 prefectures. Japan is a long, thin archipelago with its longest axis oriented from north to south. The climate of Japan is predominantly temperate, but varies greatly from north to south under the influence of the monsoon. Each number corresponds to the prefecture information presented in Tables S1, S2, S3.

### Anatomical Data

**Figure 2:**
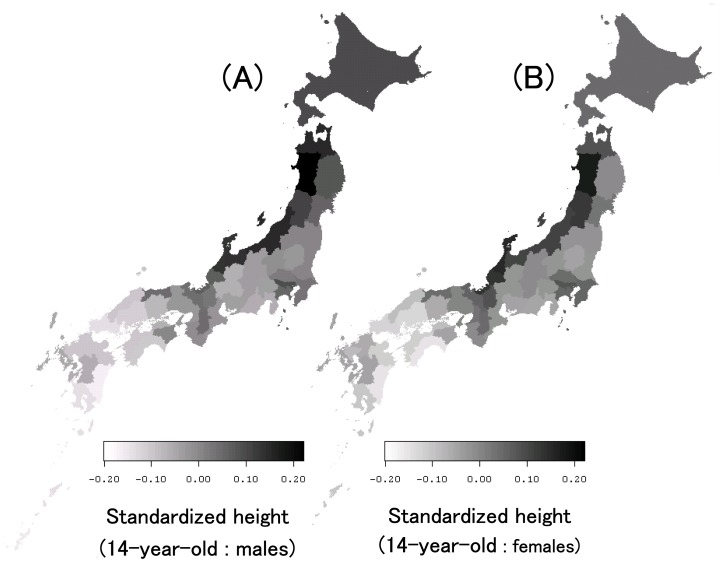
Distribution map of standardized height of Japanese youth. Distribution map of 13-year (1996 to 2008) average of the standardized height of (A) 14-year-old males and (B) 14-year-old females in each prefecture. The body height of Japanese youth tends to be larger in northern prefectures.

Prefecture-level data regarding the height of youths were collected from the reports of the School Health Examination Surveys that had been conducted from 1996 to 2008. The School Health Examination Survey is conducted by the Ministry of Education, and it collects data to determine the average values of body height categorized by sex and age (5 to 17 years) for each of the 47 prefectures in Japan [63]. A stratified two-stage sampling is used for the survey of physical condition, and a stratified cluster sampling is applied for the survey of health conditions. The 2008 survey covered approximately 7,800 schools and included approximately 700,000 pupils and students in the physical condition survey [63]. The sample size and original profile of height and weight data are shown in this database. The data collected were standardized and 13-year (1996 to 2008) averages of the standardized values were recalculated for each sex and age category to eliminate annual fluctuations in values by using the following formula:
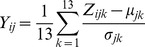
(1)where the *i* ^th^ is the prefecture; *j* ^th^ is the group (defined by age and sex); *k* ^th^ is the year (1996 to 2008); *Z_ij_* is prefecture data; *Y_ij_* is standardized height over the 13-year-period for each prefecture and gender standardized by mean; *µ_jk_* is the national average based on analysis of individual data obtained from the survey reports; and *σ_jk_* is the national-level standard deviation based on analysis of individual data regarding age and gender across Japan in year *k* obtained from the survey reports [63]. The standardized data for each prefecture are listed in Tables S1 and S2. Figure 2, which shows a map of the distribution of the standardized height of 14-year-old Japanese males and females averaged over the study’s 13-year-period, shows that the height of Japanese youth tends to be greater in the northern areas along the Japan Sea.

### Climatic Data

In this study, we calculated the 13-year-average of body size before considering the period of the climatic data that corresponded to the physical data. However, as it is uncertain whether the effects of climate remain constant throughout the growth stages, use of the climatic mean from birth until the current age of each cohort may not have been useful in this analysis. Therefore, the analysis was performed using the climatic normal, defined as the state of normal weather conditions as calculated over a 30-year period. To improve the accuracy of the values for precise analysis, mesh climatic data [12], which consist of climatic normal data, were obtained from the Japanese Meteorological Agency for analysis. Specifically, the mesh climatic dataset includes monthly averages of temperature, solar radiation, sunshine duration, precipitation, and snow cover for 1-km mesh areas, as estimated by the average of observations made from 1971 to 2000, with the climatic values in meshes with observing stations changed to observed values [12].

**Figure 3:**
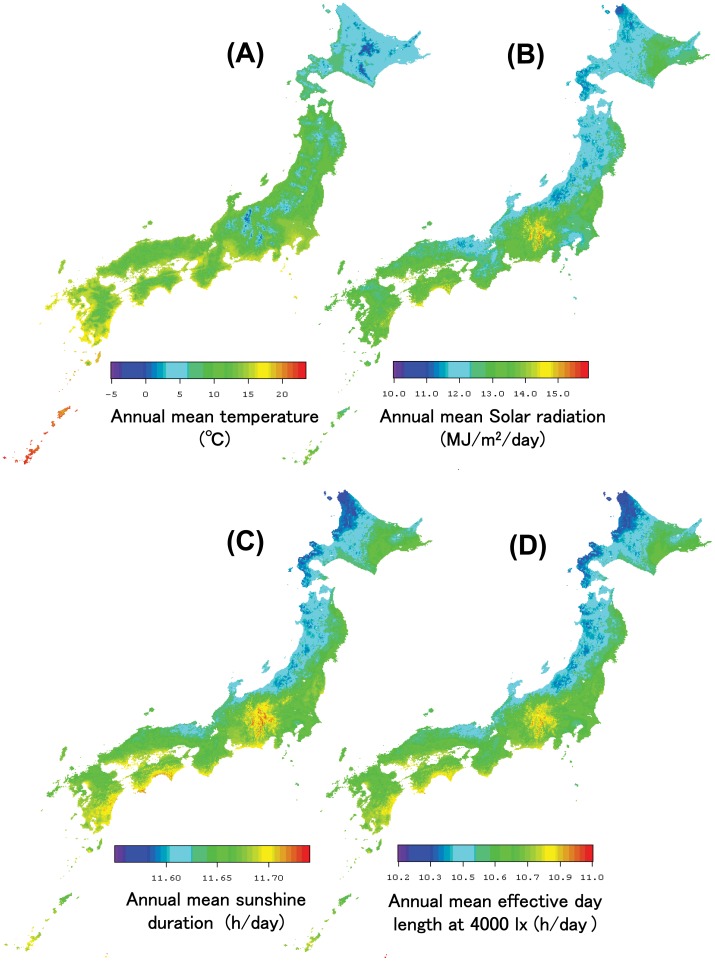
Distribution map of climatic variables in Japan. Mesh climatic data of 380,000 one-kilometer mesh areas have been shown. The fill in the mesh areas indicates (A) annual mean temperature, (B) annual mean solar radiation, (C) annual total sunshine duration, and (D) effective day length at 4000 lx. Annual mean temperature tends to be relatively low in Northern Japan, while annual mean solar radiation, annual total sunshine duration, and effective day length at 4000 lx tend to be relatively low in the northern areas along the Japan Sea.

The values for annual mean temperature, annual mean solar radiation, and annual mean sunshine duration were used in the analysis. A root mean square error between annual mean temperature in a mesh climatic value and an observed value was estimated at approximately 0.5°C in all regions of Japan [12]. The estimated relative error of annual mean global solar radiation was estimated to be less than 14% [12]. Effective day length (the duration of photoperiod exceeding the threshold of light intensity) at each 1000 lx between 1000 and 10000 lx was calculated using the following empirical model [64]–[66]:
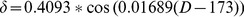
(2)

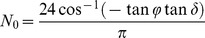
(3)where the *N*_0_ is the monthly average of daily possible sunshine duration (h/day), *D* is the Julian day of the year in the middle of a month, *φ* is the latitude (rad), and *δ* is the solar decimation (rad). When the threshold of light intensity is more than 10000 lx, the following formula can be applied [66]:
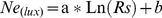
(4)

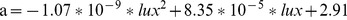
(5)

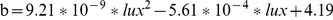
(6)where *Ne* is the monthly average of effective day length (h/day), *lux* is the arbitrary light intensity whose photoperiod is to be determined, and *Rs* is the monthly average of global solar radiation (MJ/m^2^/day). At light intensities less than 10000 lx, the following formula can be applied [66]:
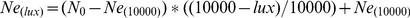
(7)where Ne(10000) is the monthly average of effective day length at a light intensity of 10000 lx (h/day). Equation (7) shows the linear interpolation between the possible sunshine duration and the monthly average of effective day length at a light intensity of 10000 lx. These formulas can be used to estimate the monthly mean effective day length at any light intensity with a high level of accuracy (less than ±10% relative error) by using solar radiation data. In this study, we used the 30-year-averages of monthly mean global solar radiation data, reported as mesh climatic data, as input values instead of observed values to eliminate fluctuations in the values. However, this assumption cannot introduce an error so large that it changes the ordinal relationship among the prefecture-based climate values. Figure 3, which shows the distribution of climatic factors in Japan, corresponds to the Japanese mesh climatic data map of 380,000 one-kilometer mesh areas, in which the fill in the mesh areas indicates (A) annual mean temperature (°C), (B) annual mean solar radiation (MJ/day), (C) annual mean sunshine duration (h/day), and (D) annual mean effective day length at 4000 lx (h/day). Annual mean temperature tends to be relatively low in Northern Japan, while annual mean solar radiation, sunshine duration, and effective day length tend to be low in the northern areas along the Japan Sea.

**Figure 4:**
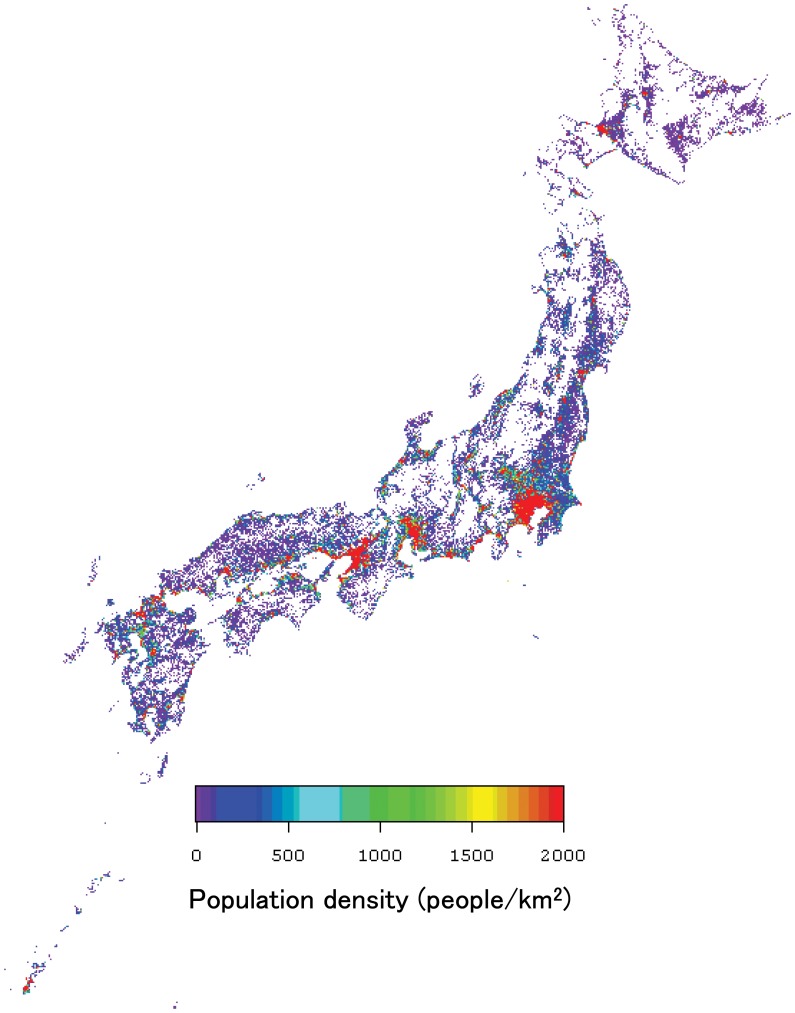
Distribution map of mesh population data in Japan. Mesh population data of 380,000 one-kilometer mesh areas has been shown. These data were derived from the results of the Population Census of 2005 and include population information by age and sex. The white areas indicate that there are no inhabitants.

Since many meshes (regions) of Japan have no inhabitants, it is undesirable to obtain an average climatic value for each prefecture by calculating a simple average of the mesh values for each prefecture. In short, there is a scale discrepancy problem between high-resolution climatic data and prefecture-level anatomical data. Therefore, 2005 Mesh Population Data [67], which were compiled from the results of the 2005 Population Census and produced under the same code and standards of the mesh climatic data, were used to calculate population-weighted average climatic values for each prefecture. Figure 4, which shows the distribution of population density (people/km^2^) in Japan, corresponds to the Japanese Mesh Population Data map of 380,000 one-kilometer mesh areas. The white areas indicate that there are no inhabitants. To consider differences in age composition, the percentages of the population aged ≤15 years in each municipality [68] were used to calculate weighted average climatic values, with the population-weighted average climatic value in each prefecture (*Cavg*) defined using the following formula:
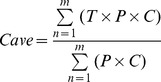
(8)where *m* is number of meshes in each prefecture, *T* is the climatic value in the mesh, *P* is the population density in the mesh, and *C* is the percentage of the population aged ≤15 years in the mesh.

These analyses were performed using GIS. GIS is a system designed to capture, store, manipulate, analyze, and present all types of geographical data. By using GIS, we can perform accurate spatial analysis. The data, in the form of ASCII text files, were manipulated by the UNIX commands “awk” [69]. Fortner TRANSFORM version 3.3 (Fortner Software, VA USA) [70] was used for drawing maps.

The population-weighted climatic value for each prefecture, rather than the average of the observed values in the capital of each prefecture, can be reasonably used to represent the average value in each prefecture. The population-weighted averaged climatic values derived from the mesh data of each prefecture are listed in Table S3.

### Data Analysis

Correlation analysis was performed using standardized body size data and mesh climatic data for all 47 prefectures, with partial correlations calculated as needed. The relationship between height and climatic factors was further explored by performing multiple linear regression modeling and using a stepwise procedure to identify important predictors of height. The potential inclusion of variables in a model was limited to those factors that improved the fit of the model at a significance level of F in = 4.0, with the criterion for retaining a variable in a model set at a significance level of F out = 2.0. All explanatory variables competed to enter the models, and no variable was forced into the models. Backward elimination procedures for model selection were used to verify the appropriateness of the variable-selection process. All statistical analyses were performed using R version 2.7.1 [71].

## Supporting Information

Table S1 **Standardized height of 8- to 17-year-old Japanese youth in each prefecture averaged over a 13-year period (1996 to 2008).**
(PDF)

Table S2 **Standardized weight of 8- to 17-year-old Japanese youth in each prefecture averaged over a 13-year period (1996 to 2008).**
(PDF)

Table S3 **Population-weighted averaged climatic values derived from mesh climatic data in each prefecture.**
(PDF)
