# Supplementary material for: Geographical Distribution of Adolescent Body Height with Respect to Effective Day Length in Japan: An Ecological Analysis
Source: PLoS One. 2012 Dec 5;7(12):e50994. doi: 10.1371/journal.pone.0050994 (PMC3515496; doi:10.1371/journal.pone.0050994)
Supplement: Table S1 — Standardized height of 8- to 17-year-old Japanese youth in each prefecture averaged over a 13-year period (1996 to 2008). (PDF) [file pone.0050994.s001.pdf]

Table S1.
Standardized height in each prefecture (averaged over a 13-year period from 1996 to 2008, 8- to 12-year-old: Males)

		Male									
No.	Prefecture	8	(SE)	9	(SE)	10	(SE)	11	(SE)	12	(SE)
1	Hokkaido	0.074	(0.038)	0.099	(0.036)	0.117	(0.042)	0.089	(0.057)	0.140	(0.032)
2	Aomori	0.187	(0.032)	0.199	(0.036)	0.227	(0.045)	0.234	(0.064)	0.211	(0.037)
3	Iwate	0.129	(0.056)	0.134	(0.038)	0.144	(0.060)	0.135	(0.031)	0.114	(0.039)
4	Miyagi	0.088	(0.055)	0.121	(0.052)	0.111	(0.042)	0.100	(0.045)	0.099	(0.058)
5	Akita	0.245	(0.042)	0.247	(0.040)	0.263	(0.053)	0.246	(0.049)	0.246	(0.041)
6	Yamagata	0.125	(0.063)	0.129	(0.057)	0.144	(0.049)	0.158	(0.057)	0.127	(0.041)
7	Fukushima	0.030	(0.036)	0.071	(0.039)	0.075	(0.045)	0.067	(0.050)	0.041	(0.043)
8	Ibaraki	0.062	(0.051)	0.054	(0.034)	0.031	(0.035)	0.031	(0.041)	0.033	(0.037)
9	Tochigi	0.010	(0.044)	-0.013	(0.049)	0.001	(0.038)	0.004	(0.055)	-0.004	(0.038)
10	Gunma	0.023	(0.053)	-0.005	(0.052)	0.012	(0.036)	0.017	(0.037)	-0.011	(0.028)
11	Saitama	0.007	(0.035)	0.024	(0.057)	-0.003	(0.033)	-0.005	(0.046)	-0.009	(0.050)
12	Chiba	0.084	(0.038)	0.055	(0.025)	0.046	(0.048)	0.028	(0.030)	0.036	(0.035)
13	Tokyo	0.074	(0.044)	0.052	(0.049)	0.049	(0.038)	0.057	(0.039)	0.061	(0.045)
14	Kanagawa	0.033	(0.046)	0.013	(0.048)	0.042	(0.037)	0.030	(0.051)	0.030	(0.045)
15	Niigata	0.136	(0.045)	0.143	(0.043)	0.137	(0.031)	0.118	(0.052)	0.131	(0.036)
16	Toyama	0.119	(0.049)	0.093	(0.042)	0.146	(0.057)	0.118	(0.036)	0.123	(0.039)
17	Ishikawa	0.060	(0.067)	0.069	(0.039)	0.064	(0.062)	0.093	(0.048)	0.073	(0.038)
18	Fukui	0.058	(0.054)	0.055	(0.051)	0.055	(0.049)	0.046	(0.048)	0.023	(0.036)
19	Yamanashi	-0.003	(0.041)	-0.020	(0.044)	-0.007	(0.047)	-0.030	(0.063)	-0.056	(0.056)
20	Nagano	-0.003	(0.038)	-0.028	(0.057)	-0.009	(0.042)	-0.039	(0.053)	-0.043	(0.028)
21	Gifu	-0.044	(0.050)	-0.030	(0.039)	-0.035	(0.064)	-0.036	(0.050)	-0.043	(0.034)
22	Shizuoka	-0.085	(0.049)	-0.066	(0.045)	-0.077	(0.044)	-0.072	(0.055)	-0.066	(0.039)
23	Aich	-0.064	(0.050)	-0.089	(0.049)	-0.064	(0.032)	-0.061	(0.029)	-0.043	(0.025)
24	Mie	-0.038	(0.031)	-0.042	(0.039)	-0.044	(0.057)	-0.019	(0.046)	-0.026	(0.037)
25	Shiga	-0.006	(0.052)	0.005	(0.039)	-0.029	(0.027)	-0.039	(0.042)	-0.060	(0.051)
26	Kyoto	0.000	(0.045)	0.005	(0.046)	0.014	(0.036)	-0.009	(0.050)	-0.011	(0.031)
27	Osaka	-0.047	(0.049)	-0.023	(0.044)	-0.049	(0.052)	-0.041	(0.064)	-0.035	(0.032)
28	Hyogo	-0.016	(0.049)	-0.036	(0.052)	-0.032	(0.049)	-0.037	(0.047)	-0.039	(0.032)
29	Nara	0.006	(0.037)	0.020	(0.048)	-0.009	(0.065)	0.019	(0.056)	0.012	(0.051)
30	Wakayama	-0.042	(0.039)	-0.003	(0.056)	-0.050	(0.069)	-0.038	(0.020)	-0.059	(0.042)
31	Tottori	0.016	(0.043)	0.013	(0.052)	0.040	(0.035)	0.044	(0.058)	0.005	(0.027)
32	Shimane	-0.129	(0.040)	-0.117	(0.051)	-0.135	(0.055)	-0.117	(0.037)	-0.098	(0.036)
33	Okayama	-0.085	(0.048)	-0.086	(0.058)	-0.076	(0.041)	-0.053	(0.053)	-0.061	(0.051)
34	Hiroshima	-0.105	(0.052)	-0.082	(0.053)	-0.097	(0.071)	-0.095	(0.049)	-0.108	(0.029)
35	Yamaguchi	-0.122	(0.052)	-0.124	(0.051)	-0.109	(0.054)	-0.116	(0.038)	-0.131	(0.037)
36	Tokushima	0.014	(0.054)	-0.005	(0.051)	0.029	(0.049)	0.065	(0.073)	0.032	(0.034)
37	Kagawa	-0.057	(0.037)	-0.047	(0.042)	-0.032	(0.036)	-0.010	(0.052)	-0.014	(0.035)
38	Ehime	-0.077	(0.054)	-0.103	(0.052)	-0.087	(0.044)	-0.084	(0.045)	-0.052	(0.033)
39	Kochi	-0.101	(0.052)	-0.093	(0.045)	-0.107	(0.042)	-0.072	(0.053)	-0.061	(0.053)
40	Fukuoka	-0.072	(0.048)	-0.059	(0.057)	-0.067	(0.054)	-0.076	(0.052)	-0.062	(0.047)
41	Saga	-0.054	(0.048)	-0.073	(0.052)	-0.069	(0.047)	-0.066	(0.036)	-0.098	(0.049)
42	Nagasaki	-0.080	(0.052)	-0.062	(0.062)	-0.081	(0.054)	-0.040	(0.053)	-0.099	(0.039)
43	Kumamoto	-0.023	(0.040)	-0.026	(0.065)	-0.040	(0.051)	-0.020	(0.046)	-0.018	(0.040)
44	Oita	-0.081	(0.033)	-0.098	(0.058)	-0.092	(0.056)	-0.077	(0.039)	-0.070	(0.025)
45	Miyazaki	-0.087	(0.040)	-0.092	(0.073)	-0.089	(0.055)	-0.066	(0.054)	-0.131	(0.044)
46	Kagoshima	-0.145	(0.062)	-0.137	(0.041)	-0.149	(0.062)	-0.135	(0.046)	-0.116	(0.053)
47	Okinawa	-0.251	(0.049)	-0.250	(0.049)	-0.217	(0.056)	-0.148	(0.041)	-0.089	(0.040)

Table S1.
Standardized height in each prefecture (averaged over a 13-year period from 1996 to 2008, 13- to 17-year-old: Males)

		Male									
No.	Prefecture	13	(SE)	14	(SE)	15	(SE)	16	(SE)	17	(SE)
1	Hokkaido	0.134	(0.036)	0.100	(0.038)	0.053	(0.040)	0.064	(0.036)	0.041	(0.030)
2	Aomori	0.195	(0.034)	0.153	(0.032)	0.124	(0.053)	0.119	(0.077)	0.116	(0.072)
3	Iwate	0.103	(0.041)	0.077	(0.040)	0.027	(0.060)	0.020	(0.067)	-0.004	(0.055)
4	Miyagi	0.097	(0.029)	0.062	(0.042)	-0.021	(0.057)	0.004	(0.054)	0.004	(0.057)
5	Akita	0.233	(0.043)	0.221	(0.046)	0.176	(0.045)	0.174	(0.054)	0.169	(0.060)
6	Yamagata	0.121	(0.034)	0.109	(0.046)	0.133	(0.071)	0.123	(0.056)	0.117	(0.055)
7	Fukushima	0.030	(0.043)	0.006	(0.044)	-0.016	(0.048)	-0.017	(0.058)	-0.017	(0.057)
8	Ibaraki	0.009	(0.039)	0.009	(0.026)	0.031	(0.062)	0.009	(0.048)	0.027	(0.048)
9	Tochigi	-0.034	(0.040)	-0.025	(0.053)	-0.044	(0.034)	-0.052	(0.042)	-0.033	(0.060)
10	Gunma	-0.008	(0.035)	-0.013	(0.042)	0.016	(0.050)	0.001	(0.063)	0.000	(0.037)
11	Saitama	-0.010	(0.038)	0.022	(0.052)	0.018	(0.041)	0.049	(0.048)	0.037	(0.052)
12	Chiba	0.021	(0.032)	0.031	(0.031)	0.033	(0.051)	0.032	(0.042)	0.037	(0.036)
13	Tokyo	0.090	(0.031)	0.093	(0.043)	0.083	(0.047)	0.072	(0.034)	0.066	(0.043)
14	Kanagawa	0.025	(0.038)	0.057	(0.040)	0.055	(0.038)	0.068	(0.045)	0.100	(0.049)
15	Niigata	0.126	(0.031)	0.149	(0.028)	0.153	(0.061)	0.128	(0.040)	0.132	(0.052)
16	Toyama	0.127	(0.048)	0.137	(0.050)	0.147	(0.059)	0.122	(0.040)	0.116	(0.048)
17	Ishikawa	0.111	(0.054)	0.132	(0.044)	0.167	(0.055)	0.159	(0.061)	0.156	(0.047)
18	Fukui	0.070	(0.027)	0.070	(0.044)	0.077	(0.057)	0.087	(0.053)	0.095	(0.065)
19	Yamanashi	-0.054	(0.048)	-0.038	(0.024)	-0.038	(0.038)	0.008	(0.071)	-0.029	(0.048)
20	Nagano	-0.043	(0.052)	-0.031	(0.037)	-0.039	(0.063)	-0.018	(0.071)	0.000	(0.052)
21	Gifu	-0.059	(0.038)	-0.063	(0.044)	-0.058	(0.051)	-0.022	(0.054)	-0.025	(0.034)
22	Shizuoka	-0.059	(0.036)	-0.060	(0.057)	-0.055	(0.067)	-0.052	(0.050)	-0.052	(0.041)
23	Aich	-0.037	(0.038)	-0.041	(0.032)	-0.018	(0.050)	-0.053	(0.052)	-0.073	(0.035)
24	Mie	-0.035	(0.023)	-0.022	(0.044)	-0.029	(0.074)	0.004	(0.049)	-0.017	(0.061)
25	Shiga	-0.019	(0.033)	0.025	(0.026)	0.033	(0.037)	0.045	(0.045)	0.101	(0.035)
26	Kyoto	-0.001	(0.030)	0.041	(0.040)	0.065	(0.047)	0.078	(0.057)	0.068	(0.056)
27	Osaka	-0.020	(0.032)	-0.027	(0.051)	0.017	(0.059)	0.012	(0.028)	0.029	(0.027)
28	Hyogo	-0.055	(0.047)	-0.024	(0.031)	0.001	(0.040)	0.016	(0.047)	-0.004	(0.047)
29	Nara	0.032	(0.034)	0.044	(0.027)	0.024	(0.056)	0.040	(0.067)	0.033	(0.075)
30	Wakayama	-0.020	(0.050)	-0.005	(0.048)	-0.015	(0.059)	-0.012	(0.059)	0.020	(0.044)
31	Tottori	0.052	(0.037)	0.044	(0.039)	0.044	(0.059)	0.049	(0.050)	0.071	(0.053)
32	Shimane	-0.107	(0.036)	-0.098	(0.036)	-0.064	(0.062)	-0.053	(0.034)	-0.032	(0.042)
33	Okayama	-0.074	(0.048)	-0.080	(0.039)	-0.103	(0.065)	-0.096	(0.042)	-0.098	(0.044)
34	Hiroshima	-0.103	(0.053)	-0.111	(0.029)	-0.134	(0.040)	-0.130	(0.046)	-0.114	(0.067)
35	Yamaguchi	-0.146	(0.039)	-0.136	(0.045)	-0.119	(0.047)	-0.112	(0.049)	-0.092	(0.041)
36	Tokushima	0.024	(0.029)	0.000	(0.047)	-0.023	(0.037)	-0.040	(0.037)	-0.076	(0.045)
37	Kagawa	-0.038	(0.030)	-0.066	(0.034)	-0.089	(0.043)	-0.101	(0.047)	-0.096	(0.053)
38	Ehime	-0.081	(0.027)	-0.097	(0.057)	-0.150	(0.052)	-0.146	(0.070)	-0.126	(0.036)
39	Kochi	-0.098	(0.037)	-0.104	(0.031)	-0.132	(0.042)	-0.113	(0.073)	-0.133	(0.045)
40	Fukuoka	-0.083	(0.046)	-0.100	(0.051)	-0.102	(0.041)	-0.119	(0.052)	-0.106	(0.041)
41	Saga	-0.098	(0.036)	-0.094	(0.030)	-0.093	(0.054)	-0.080	(0.051)	-0.073	(0.072)
42	Nagasaki	-0.061	(0.031)	-0.053	(0.026)	-0.045	(0.049)	-0.070	(0.050)	-0.053	(0.057)
43	Kumamoto	-0.047	(0.042)	-0.059	(0.039)	-0.056	(0.062)	-0.068	(0.054)	-0.052	(0.058)
44	Oita	-0.086	(0.053)	-0.094	(0.031)	-0.107	(0.047)	-0.093	(0.049)	-0.105	(0.045)
45	Miyazaki	-0.137	(0.048)	-0.165	(0.056)	-0.140	(0.066)	-0.164	(0.045)	-0.142	(0.063)
46	Kagoshima	-0.114	(0.046)	-0.137	(0.040)	-0.136	(0.052)	-0.106	(0.037)	-0.089	(0.065)
47	Okinawa	-0.110	(0.037)	-0.160	(0.046)	-0.263	(0.045)	-0.285	(0.046)	-0.302	(0.042)

Table S1.
Standardized height in each prefecture (averaged over a 13-year period from 1996 to 2008, 8- to 12-year-old: Females)

		Female									
No.	Prefecture	8	(SE)	9	(SE)	10	(SE)	11	(SE)	12	(SE)
1	Hokkaido	0.053	(0.039)	0.096	(0.042)	0.106	(0.052)	0.080	(0.041)	0.080	(0.042)
2	Aomori	0.235	(0.062)	0.253	(0.059)	0.254	(0.066)	0.216	(0.029)	0.139	(0.025)
3	Iwate	0.125	(0.038)	0.151	(0.035)	0.115	(0.046)	0.084	(0.065)	0.045	(0.040)
4	Miyagi	0.104	(0.061)	0.112	(0.037)	0.116	(0.052)	0.071	(0.038)	0.060	(0.027)
5	Akita	0.228	(0.033)	0.233	(0.038)	0.221	(0.056)	0.201	(0.046)	0.203	(0.038)
6	Yamagata	0.133	(0.049)	0.138	(0.052)	0.106	(0.055)	0.110	(0.043)	0.113	(0.042)
7	Fukushima	0.082	(0.044)	0.048	(0.050)	0.071	(0.046)	0.061	(0.039)	-0.001	(0.040)
8	Ibaraki	0.029	(0.051)	0.018	(0.055)	0.028	(0.048)	0.023	(0.058)	0.006	(0.040)
9	Tochigi	0.004	(0.027)	-0.015	(0.043)	0.012	(0.051)	-0.023	(0.044)	-0.016	(0.042)
10	Gunma	-0.005	(0.049)	-0.022	(0.031)	-0.001	(0.065)	-0.012	(0.050)	-0.034	(0.036)
11	Saitama	0.031	(0.053)	0.004	(0.029)	0.003	(0.058)	0.006	(0.054)	0.017	(0.048)
12	Chiba	0.044	(0.039)	0.041	(0.065)	0.057	(0.039)	0.050	(0.049)	0.019	(0.038)
13	Tokyo	0.038	(0.041)	0.030	(0.041)	-0.002	(0.040)	0.051	(0.038)	0.073	(0.047)
14	Kanagawa	0.015	(0.044)	0.015	(0.046)	0.008	(0.048)	0.013	(0.055)	0.035	(0.034)
15	Niigata	0.146	(0.054)	0.119	(0.033)	0.102	(0.053)	0.117	(0.053)	0.117	(0.038)
16	Toyama	0.129	(0.049)	0.131	(0.042)	0.119	(0.048)	0.119	(0.030)	0.123	(0.042)
17	Ishikawa	0.096	(0.059)	0.068	(0.058)	0.074	(0.043)	0.079	(0.046)	0.118	(0.046)
18	Fukui	0.061	(0.034)	0.080	(0.042)	0.085	(0.043)	0.069	(0.049)	0.071	(0.026)
19	Yamanashi	-0.001	(0.057)	0.006	(0.046)	-0.017	(0.048)	-0.027	(0.056)	-0.040	(0.039)
20	Nagano	-0.031	(0.043)	-0.036	(0.064)	-0.049	(0.060)	-0.040	(0.041)	-0.010	(0.037)
21	Gifu	-0.045	(0.039)	-0.063	(0.047)	-0.065	(0.051)	-0.043	(0.039)	-0.032	(0.039)
22	Shizuoka	-0.094	(0.026)	-0.060	(0.052)	-0.060	(0.040)	-0.067	(0.040)	-0.066	(0.040)
23	Aich	-0.083	(0.051)	-0.088	(0.040)	-0.083	(0.046)	-0.091	(0.039)	-0.073	(0.038)
24	Mie	-0.032	(0.047)	-0.040	(0.039)	-0.060	(0.063)	-0.013	(0.045)	-0.038	(0.030)
25	Shiga	0.007	(0.058)	-0.011	(0.041)	-0.024	(0.047)	-0.001	(0.054)	0.018	(0.025)
26	Kyoto	-0.018	(0.047)	-0.001	(0.048)	-0.012	(0.057)	0.002	(0.052)	0.016	(0.029)
27	Osaka	-0.021	(0.047)	-0.041	(0.035)	-0.020	(0.040)	-0.032	(0.038)	-0.009	(0.033)
28	Hyogo	-0.035	(0.033)	0.003	(0.049)	-0.047	(0.061)	-0.030	(0.050)	-0.019	(0.028)
29	Nara	0.031	(0.041)	-0.009	(0.053)	0.010	(0.050)	-0.008	(0.045)	0.009	(0.047)
30	Wakayama	-0.035	(0.066)	-0.011	(0.060)	-0.026	(0.041)	-0.017	(0.063)	-0.028	(0.052)
31	Tottori	0.018	(0.047)	0.035	(0.048)	0.038	(0.045)	0.055	(0.044)	0.021	(0.047)
32	Shimane	-0.082	(0.071)	-0.075	(0.052)	-0.078	(0.065)	-0.083	(0.032)	-0.108	(0.050)
33	Okayama	-0.057	(0.041)	-0.065	(0.048)	-0.087	(0.050)	-0.064	(0.044)	-0.074	(0.043)
34	Hiroshima	-0.084	(0.057)	-0.100	(0.046)	-0.088	(0.063)	-0.094	(0.048)	-0.106	(0.037)
35	Yamaguchi	-0.111	(0.048)	-0.124	(0.045)	-0.119	(0.045)	-0.078	(0.066)	-0.122	(0.039)
36	Tokushima	0.001	(0.057)	0.004	(0.058)	0.024	(0.047)	0.008	(0.037)	-0.017	(0.048)
37	Kagawa	-0.064	(0.043)	-0.050	(0.034)	-0.028	(0.046)	-0.047	(0.056)	-0.062	(0.037)
38	Ehime	-0.079	(0.040)	-0.047	(0.052)	-0.062	(0.053)	-0.077	(0.043)	-0.076	(0.030)
39	Kochi	-0.088	(0.057)	-0.058	(0.052)	-0.063	(0.069)	-0.067	(0.045)	-0.074	(0.042)
40	Fukuoka	-0.026	(0.047)	-0.042	(0.053)	-0.024	(0.037)	-0.047	(0.051)	-0.052	(0.034)
41	Saga	-0.038	(0.029)	-0.021	(0.041)	-0.016	(0.052)	-0.014	(0.085)	-0.073	(0.034)
42	Nagasaki	-0.063	(0.043)	-0.035	(0.040)	-0.028	(0.065)	-0.052	(0.038)	-0.069	(0.038)
43	Kumamoto	-0.004	(0.051)	0.004	(0.034)	-0.009	(0.064)	0.012	(0.053)	-0.036	(0.056)
44	Oita	-0.074	(0.044)	-0.052	(0.066)	-0.058	(0.045)	-0.069	(0.044)	-0.088	(0.046)
45	Miyazaki	-0.062	(0.062)	-0.064	(0.059)	-0.033	(0.051)	-0.045	(0.064)	-0.118	(0.029)
46	Kagoshima	-0.121	(0.057)	-0.114	(0.060)	-0.093	(0.058)	-0.092	(0.062)	-0.087	(0.044)
47	Okinawa	-0.138	(0.049)	-0.076	(0.067)	-0.033	(0.060)	-0.068	(0.039)	-0.148	(0.043)

Table S1.
Standardized height in each prefecture (averaged over a 13-year period from 1996 to 2008, 13- to 17-year-old: Females)

		Female									
No.	Prefecture	13	(SE)	14	(SE)	15	(SE)	16	(SE)	17	(SE)
1	Hokkaido	0.067	(0.033)	0.048	(0.035)	0.051	(0.061)	0.061	(0.036)	0.032	(0.057)
2	Aomori	0.128	(0.047)	0.085	(0.043)	0.068	(0.068)	0.073	(0.047)	0.071	(0.052)
3	Iwate	0.024	(0.032)	-0.009	(0.038)	-0.043	(0.051)	-0.039	(0.052)	-0.018	(0.040)
4	Miyagi	0.033	(0.042)	0.026	(0.045)	0.013	(0.070)	-0.006	(0.048)	0.014	(0.064)
5	Akita	0.172	(0.053)	0.171	(0.045)	0.133	(0.037)	0.124	(0.051)	0.126	(0.073)
6	Yamagata	0.117	(0.037)	0.126	(0.047)	0.120	(0.051)	0.089	(0.033)	0.121	(0.060)
7	Fukushima	-0.013	(0.034)	-0.021	(0.034)	-0.032	(0.069)	-0.019	(0.047)	-0.026	(0.053)
8	Ibaraki	-0.004	(0.040)	-0.023	(0.044)	0.006	(0.031)	-0.003	(0.049)	-0.003	(0.041)
9	Tochigi	-0.028	(0.022)	-0.044	(0.031)	-0.051	(0.053)	-0.055	(0.054)	-0.049	(0.054)
10	Gunma	-0.018	(0.032)	-0.029	(0.041)	0.000	(0.050)	0.009	(0.046)	0.016	(0.045)
11	Saitama	0.026	(0.036)	0.039	(0.030)	0.035	(0.046)	0.061	(0.060)	0.028	(0.042)
12	Chiba	0.038	(0.031)	0.051	(0.045)	0.031	(0.036)	0.029	(0.048)	0.016	(0.044)
13	Tokyo	0.073	(0.033)	0.077	(0.033)	0.067	(0.050)	0.087	(0.038)	0.080	(0.056)
14	Kanagawa	0.063	(0.037)	0.051	(0.038)	0.075	(0.052)	0.080	(0.060)	0.075	(0.075)
15	Niigata	0.108	(0.039)	0.114	(0.030)	0.110	(0.054)	0.096	(0.053)	0.135	(0.042)
16	Toyama	0.131	(0.035)	0.104	(0.041)	0.104	(0.058)	0.098	(0.055)	0.134	(0.033)
17	Ishikawa	0.122	(0.035)	0.151	(0.047)	0.114	(0.035)	0.098	(0.050)	0.090	(0.054)
18	Fukui	0.115	(0.027)	0.133	(0.030)	0.122	(0.062)	0.108	(0.040)	0.111	(0.035)
19	Yamanashi	-0.034	(0.036)	-0.023	(0.026)	-0.007	(0.052)	-0.003	(0.041)	0.001	(0.038)
20	Nagano	-0.043	(0.041)	-0.007	(0.036)	-0.009	(0.040)	0.013	(0.040)	0.012	(0.047)
21	Gifu	-0.038	(0.035)	-0.025	(0.052)	-0.020	(0.044)	-0.013	(0.040)	-0.030	(0.045)
22	Shizuoka	-0.060	(0.047)	-0.054	(0.032)	-0.038	(0.064)	-0.048	(0.052)	-0.041	(0.033)
23	Aich	-0.058	(0.037)	-0.044	(0.043)	-0.043	(0.038)	-0.039	(0.035)	-0.073	(0.037)
24	Mie	-0.021	(0.024)	-0.028	(0.045)	-0.034	(0.041)	-0.025	(0.056)	-0.019	(0.054)
25	Shiga	0.070	(0.026)	0.084	(0.037)	0.066	(0.039)	0.102	(0.039)	0.125	(0.036)
26	Kyoto	0.058	(0.038)	0.058	(0.044)	0.081	(0.053)	0.077	(0.055)	0.066	(0.055)
27	Osaka	0.026	(0.033)	0.031	(0.038)	0.059	(0.049)	0.042	(0.043)	0.036	(0.059)
28	Hyogo	-0.011	(0.030)	-0.002	(0.033)	0.003	(0.050)	0.003	(0.038)	0.042	(0.053)
29	Nara	0.040	(0.059)	0.047	(0.050)	0.037	(0.028)	0.032	(0.055)	0.038	(0.070)
30	Wakayama	0.003	(0.042)	-0.004	(0.044)	-0.025	(0.067)	0.016	(0.064)	0.007	(0.058)
31	Tottori	0.034	(0.043)	0.039	(0.037)	0.041	(0.048)	0.032	(0.061)	0.044	(0.048)
32	Shimane	-0.088	(0.043)	-0.091	(0.037)	-0.088	(0.075)	-0.102	(0.051)	-0.060	(0.038)
33	Okayama	-0.083	(0.034)	-0.059	(0.029)	-0.097	(0.037)	-0.109	(0.055)	-0.115	(0.052)
34	Hiroshima	-0.114	(0.041)	-0.133	(0.048)	-0.098	(0.052)	-0.129	(0.060)	-0.108	(0.043)
35	Yamaguchi	-0.138	(0.037)	-0.126	(0.042)	-0.082	(0.055)	-0.096	(0.055)	-0.065	(0.045)
36	Tokushima	-0.033	(0.025)	-0.044	(0.043)	-0.060	(0.048)	-0.030	(0.052)	-0.058	(0.052)
37	Kagawa	-0.103	(0.024)	-0.092	(0.048)	-0.104	(0.057)	-0.096	(0.043)	-0.082	(0.030)
38	Ehime	-0.121	(0.027)	-0.118	(0.041)	-0.167	(0.040)	-0.140	(0.035)	-0.118	(0.051)
39	Kochi	-0.130	(0.022)	-0.148	(0.038)	-0.155	(0.053)	-0.150	(0.059)	-0.151	(0.057)
40	Fukuoka	-0.038	(0.044)	-0.057	(0.026)	-0.081	(0.044)	-0.097	(0.039)	-0.105	(0.061)
41	Saga	-0.050	(0.044)	-0.086	(0.039)	-0.100	(0.066)	-0.058	(0.054)	-0.074	(0.067)
42	Nagasaki	-0.023	(0.053)	-0.045	(0.030)	-0.068	(0.051)	-0.026	(0.044)	-0.042	(0.067)
43	Kumamoto	-0.031	(0.035)	-0.044	(0.029)	-0.035	(0.049)	-0.020	(0.053)	-0.002	(0.049)
44	Oita	-0.117	(0.041)	-0.120	(0.033)	-0.113	(0.041)	-0.144	(0.045)	-0.121	(0.066)
45	Miyazaki	-0.151	(0.030)	-0.152	(0.029)	-0.122	(0.069)	-0.166	(0.043)	-0.176	(0.031)
46	Kagoshima	-0.086	(0.045)	-0.104	(0.039)	-0.108	(0.041)	-0.105	(0.038)	-0.102	(0.040)
47	Okinawa	-0.269	(0.052)	-0.305	(0.052)	-0.322	(0.056)	-0.324	(0.073)	-0.311	(0.023)
