# Supplementary material for: Geographical Distribution of Adolescent Body Height with Respect to Effective Day Length in Japan: An Ecological Analysis
Source: PLoS One. 2012 Dec 5;7(12):e50994. doi: 10.1371/journal.pone.0050994 (PMC3515496; doi:10.1371/journal.pone.0050994)
Supplement: Table S2 — Standardized weight of 8- to 17-year-old Japanese youth in each prefecture averaged over a 13-year period (1996 to 2008). (PDF) [file pone.0050994.s002.pdf]

Table S2.
Standardized weight in each prefecture (averaged over a 13-year period from 1996 to 2008, 8- to 12-year-old: Males)

		Male									
No.	Prefecture	8	(SE)	9	(SE)	10	(SE)	11	(SE)	12	(SE)
1	Hokkaido	0.181	(0.053)	0.205	(0.046)	0.194	(0.054)	0.177	(0.069)	0.201	(0.031)
2	Aomori	0.306	(0.062)	0.303	(0.071)	0.289	(0.059)	0.294	(0.059)	0.309	(0.046)
3	Iwate	0.195	(0.057)	0.201	(0.046)	0.199	(0.055)	0.210	(0.035)	0.190	(0.045)
4	Miyagi	0.160	(0.056)	0.192	(0.066)	0.154	(0.053)	0.154	(0.050)	0.162	(0.046)
5	Akita	0.276	(0.063)	0.297	(0.049)	0.262	(0.071)	0.221	(0.068)	0.245	(0.021)
6	Yamagata	0.174	(0.054)	0.155	(0.075)	0.182	(0.064)	0.181	(0.060)	0.139	(0.033)
7	Fukushima	0.132	(0.057)	0.152	(0.042)	0.142	(0.070)	0.115	(0.050)	0.098	(0.042)
8	Ibaraki	0.129	(0.052)	0.106	(0.042)	0.081	(0.065)	0.085	(0.054)	0.085	(0.048)
9	Tochigi	0.132	(0.046)	0.079	(0.053)	0.097	(0.057)	0.085	(0.048)	0.095	(0.040)
10	Gunma	0.049	(0.053)	0.045	(0.048)	0.039	(0.041)	0.055	(0.038)	0.039	(0.049)
11	Saitama	-0.017	(0.037)	0.013	(0.053)	-0.002	(0.048)	-0.029	(0.044)	-0.025	(0.045)
12	Chiba	0.030	(0.043)	0.022	(0.048)	0.042	(0.049)	0.025	(0.031)	0.008	(0.035)
13	Tokyo	0.002	(0.049)	0.012	(0.056)	0.016	(0.038)	0.041	(0.045)	0.039	(0.045)
14	Kanagawa	-0.021	(0.041)	-0.030	(0.050)	-0.009	(0.035)	-0.020	(0.055)	-0.013	(0.040)
15	Niigata	0.063	(0.054)	0.088	(0.062)	0.062	(0.047)	0.029	(0.066)	0.043	(0.032)
16	Toyama	0.074	(0.054)	0.064	(0.036)	0.079	(0.060)	0.053	(0.037)	0.069	(0.030)
17	Ishikawa	0.005	(0.045)	0.042	(0.039)	0.025	(0.068)	0.041	(0.053)	0.027	(0.048)
18	Fukui	0.019	(0.044)	-0.013	(0.046)	-0.002	(0.039)	-0.016	(0.053)	-0.022	(0.038)
19	Yamanashi	-0.011	(0.052)	-0.008	(0.057)	0.012	(0.048)	-0.033	(0.052)	-0.013	(0.064)
20	Nagano	-0.072	(0.038)	-0.092	(0.038)	-0.059	(0.044)	-0.070	(0.044)	-0.052	(0.031)
21	Gifu	-0.049	(0.057)	-0.036	(0.038)	-0.051	(0.046)	-0.071	(0.053)	-0.076	(0.038)
22	Shizuoka	-0.091	(0.045)	-0.080	(0.034)	-0.081	(0.046)	-0.077	(0.044)	-0.085	(0.033)
23	Aich	-0.056	(0.047)	-0.085	(0.037)	-0.065	(0.038)	-0.058	(0.038)	-0.057	(0.036)
24	Mie	-0.030	(0.053)	-0.047	(0.039)	-0.030	(0.036)	-0.020	(0.039)	-0.040	(0.039)
25	Shiga	-0.074	(0.051)	-0.078	(0.049)	-0.115	(0.031)	-0.116	(0.059)	-0.128	(0.039)
26	Kyoto	-0.110	(0.042)	-0.077	(0.037)	-0.083	(0.051)	-0.090	(0.052)	-0.070	(0.045)
27	Osaka	-0.049	(0.046)	-0.028	(0.047)	-0.048	(0.043)	-0.043	(0.049)	-0.042	(0.037)
28	Hyogo	-0.073	(0.046)	-0.098	(0.047)	-0.100	(0.042)	-0.065	(0.058)	-0.072	(0.037)
29	Nara	-0.038	(0.037)	-0.041	(0.043)	-0.028	(0.048)	-0.010	(0.047)	-0.012	(0.046)
30	Wakayama	-0.023	(0.053)	-0.009	(0.057)	-0.026	(0.066)	-0.006	(0.044)	-0.009	(0.029)
31	Tottori	-0.028	(0.040)	-0.057	(0.041)	-0.040	(0.052)	-0.045	(0.035)	-0.080	(0.029)
32	Shimane	-0.104	(0.043)	-0.117	(0.049)	-0.150	(0.052)	-0.147	(0.028)	-0.147	(0.029)
33	Okayama	-0.080	(0.029)	-0.104	(0.047)	-0.072	(0.060)	-0.054	(0.064)	-0.077	(0.042)
34	Hiroshima	-0.084	(0.030)	-0.054	(0.056)	-0.062	(0.052)	-0.076	(0.047)	-0.067	(0.033)
35	Yamaguchi	-0.110	(0.051)	-0.102	(0.045)	-0.108	(0.047)	-0.120	(0.044)	-0.121	(0.045)
36	Tokushima	0.111	(0.034)	0.088	(0.051)	0.125	(0.071)	0.139	(0.074)	0.139	(0.031)
37	Kagawa	-0.040	(0.035)	0.002	(0.033)	0.012	(0.046)	0.003	(0.061)	0.063	(0.036)
38	Ehime	-0.045	(0.053)	-0.048	(0.049)	-0.053	(0.069)	-0.052	(0.033)	-0.020	(0.038)
39	Kochi	-0.071	(0.044)	-0.055	(0.051)	-0.049	(0.064)	-0.035	(0.057)	-0.006	(0.058)
40	Fukuoka	-0.060	(0.043)	-0.076	(0.059)	-0.072	(0.048)	-0.073	(0.045)	-0.084	(0.046)
41	Saga	-0.048	(0.038)	-0.074	(0.053)	-0.059	(0.059)	-0.080	(0.056)	-0.064	(0.048)
42	Nagasaki	-0.068	(0.054)	-0.067	(0.063)	-0.096	(0.054)	-0.072	(0.060)	-0.120	(0.041)
43	Kumamoto	0.012	(0.054)	0.018	(0.075)	-0.040	(0.054)	-0.035	(0.063)	-0.053	(0.045)
44	Oita	-0.009	(0.040)	-0.029	(0.063)	-0.026	(0.056)	-0.046	(0.058)	-0.007	(0.030)
45	Miyazaki	-0.024	(0.065)	-0.040	(0.062)	-0.051	(0.059)	-0.027	(0.065)	-0.063	(0.060)
46	Kagoshima	-0.074	(0.064)	-0.079	(0.034)	-0.093	(0.052)	-0.091	(0.059)	-0.106	(0.058)
47	Okinawa	-0.063	(0.041)	-0.082	(0.073)	-0.087	(0.059)	-0.039	(0.046)	-0.016	(0.032)

Table S2.
Standardized weight in each prefecture (averaged over a 13-year period from 1996 to 2008, 13- to 17-year-old: Males)

		Male									
No.	Prefecture	13	(SE)	14	(SE)	15	(SE)	16	(SE)	17	(SE)
1	Hokkaido	0.199	(0.043)	0.157	(0.036)	0.095	(0.051)	0.122	(0.039)	0.113	(0.078)
2	Aomori	0.272	(0.054)	0.258	(0.028)	0.212	(0.033)	0.251	(0.077)	0.240	(0.079)
3	Iwate	0.136	(0.034)	0.143	(0.056)	0.133	(0.050)	0.121	(0.067)	0.131	(0.047)
4	Miyagi	0.136	(0.034)	0.116	(0.050)	0.110	(0.050)	0.110	(0.054)	0.111	(0.060)
5	Akita	0.225	(0.054)	0.195	(0.052)	0.201	(0.058)	0.245	(0.054)	0.228	(0.050)
6	Yamagata	0.110	(0.032)	0.125	(0.035)	0.127	(0.072)	0.132	(0.056)	0.126	(0.046)
7	Fukushima	0.110	(0.060)	0.069	(0.041)	0.111	(0.066)	0.085	(0.058)	0.086	(0.048)
8	Ibaraki	0.043	(0.023)	0.064	(0.039)	0.055	(0.051)	0.064	(0.048)	0.037	(0.040)
9	Tochigi	0.034	(0.037)	0.055	(0.048)	0.077	(0.053)	0.083	(0.042)	0.087	(0.079)
10	Gunma	0.036	(0.034)	0.035	(0.025)	0.071	(0.071)	0.046	(0.063)	0.037	(0.075)
11	Saitama	-0.039	(0.039)	-0.029	(0.041)	-0.014	(0.046)	0.005	(0.048)	-0.022	(0.059)
12	Chiba	-0.014	(0.029)	0.002	(0.032)	0.047	(0.066)	0.018	(0.042)	0.019	(0.051)
13	Tokyo	0.082	(0.039)	0.056	(0.036)	0.046	(0.040)	0.026	(0.034)	0.031	(0.053)
14	Kanagawa	-0.020	(0.035)	0.014	(0.025)	-0.005	(0.045)	-0.012	(0.045)	-0.030	(0.051)
15	Niigata	0.052	(0.042)	0.050	(0.044)	0.044	(0.033)	0.029	(0.040)	0.050	(0.043)
16	Toyama	0.065	(0.032)	0.073	(0.053)	0.091	(0.040)	0.050	(0.040)	0.047	(0.059)
17	Ishikawa	0.019	(0.043)	0.030	(0.043)	0.074	(0.043)	0.079	(0.061)	0.078	(0.049)
18	Fukui	-0.023	(0.041)	-0.012	(0.034)	0.030	(0.045)	0.057	(0.053)	0.016	(0.034)
19	Yamanashi	0.000	(0.046)	0.001	(0.029)	0.019	(0.037)	0.027	(0.071)	0.032	(0.048)
20	Nagano	-0.050	(0.038)	-0.054	(0.038)	-0.046	(0.056)	-0.025	(0.071)	-0.010	(0.042)
21	Gifu	-0.064	(0.043)	-0.075	(0.034)	-0.074	(0.034)	-0.074	(0.054)	-0.091	(0.046)
22	Shizuoka	-0.099	(0.044)	-0.094	(0.053)	-0.082	(0.058)	-0.095	(0.050)	-0.086	(0.039)
23	Aich	-0.035	(0.043)	-0.027	(0.047)	-0.043	(0.050)	-0.063	(0.052)	-0.069	(0.038)
24	Mie	-0.058	(0.033)	-0.057	(0.032)	-0.033	(0.072)	-0.042	(0.049)	-0.064	(0.040)
25	Shiga	-0.080	(0.030)	-0.065	(0.035)	-0.047	(0.064)	-0.033	(0.045)	-0.011	(0.040)
26	Kyoto	-0.075	(0.043)	-0.043	(0.049)	-0.079	(0.059)	-0.049	(0.057)	-0.052	(0.048)
27	Osaka	0.002	(0.026)	0.002	(0.056)	-0.012	(0.068)	-0.015	(0.028)	0.012	(0.049)
28	Hyogo	-0.093	(0.036)	-0.066	(0.029)	-0.067	(0.050)	-0.053	(0.047)	-0.039	(0.044)
29	Nara	-0.007	(0.035)	0.007	(0.031)	-0.011	(0.039)	-0.022	(0.067)	-0.034	(0.064)
30	Wakayama	0.038	(0.049)	0.032	(0.037)	0.026	(0.032)	-0.006	(0.059)	0.020	(0.050)
31	Tottori	-0.017	(0.030)	-0.052	(0.045)	-0.068	(0.055)	-0.061	(0.050)	-0.074	(0.042)
32	Shimane	-0.151	(0.034)	-0.140	(0.041)	-0.126	(0.036)	-0.095	(0.034)	-0.056	(0.036)
33	Okayama	-0.066	(0.048)	-0.064	(0.047)	-0.060	(0.049)	-0.072	(0.042)	-0.050	(0.069)
34	Hiroshima	-0.065	(0.050)	-0.052	(0.033)	-0.098	(0.038)	-0.094	(0.046)	-0.076	(0.040)
35	Yamaguchi	-0.163	(0.047)	-0.152	(0.027)	-0.154	(0.045)	-0.156	(0.049)	-0.142	(0.037)
36	Tokushima	0.136	(0.027)	0.128	(0.046)	0.126	(0.063)	0.123	(0.037)	0.051	(0.052)
37	Kagawa	0.024	(0.042)	0.033	(0.015)	0.035	(0.030)	0.020	(0.047)	-0.001	(0.046)
38	Ehime	-0.034	(0.030)	-0.055	(0.038)	-0.055	(0.061)	-0.049	(0.070)	-0.041	(0.055)
39	Kochi	-0.048	(0.027)	-0.053	(0.042)	-0.079	(0.045)	-0.056	(0.073)	-0.082	(0.059)
40	Fukuoka	-0.087	(0.036)	-0.094	(0.050)	-0.073	(0.054)	-0.074	(0.052)	-0.077	(0.049)
41	Saga	-0.083	(0.054)	-0.089	(0.033)	-0.059	(0.066)	-0.050	(0.051)	-0.036	(0.050)
42	Nagasaki	-0.098	(0.035)	-0.100	(0.040)	-0.049	(0.041)	-0.048	(0.050)	-0.039	(0.053)
43	Kumamoto	-0.056	(0.040)	-0.058	(0.048)	-0.071	(0.058)	-0.051	(0.054)	-0.055	(0.053)
44	Oita	-0.012	(0.044)	-0.008	(0.036)	0.004	(0.048)	0.033	(0.049)	0.021	(0.051)
45	Miyazaki	-0.084	(0.053)	-0.095	(0.052)	-0.051	(0.051)	-0.085	(0.045)	-0.068	(0.074)
46	Kagoshima	-0.099	(0.042)	-0.105	(0.055)	-0.079	(0.045)	-0.040	(0.037)	-0.039	(0.050)
47	Okinawa	-0.026	(0.054)	-0.045	(0.050)	-0.107	(0.027)	-0.135	(0.046)	-0.122	(0.055)

Table S2.
Standardized weight in each prefecture (averaged over a 13-year period from 1996 to 2008, 8- to 12-year-old: Females)

		Female									
No.	Prefecture	8	(SE)	9	(SE)	10	(SE)	11	(SE)	12	(SE)
1	Hokkaido	0.144	(0.065)	0.143	(0.059)	0.176	(0.063)	0.126	(0.058)	0.112	(0.031)
2	Aomori	0.312	(0.078)	0.319	(0.072)	0.314	(0.072)	0.236	(0.051)	0.238	(0.028)
3	Iwate	0.190	(0.055)	0.237	(0.060)	0.213	(0.050)	0.191	(0.060)	0.173	(0.043)
4	Miyagi	0.175	(0.047)	0.168	(0.059)	0.181	(0.049)	0.153	(0.051)	0.154	(0.040)
5	Akita	0.250	(0.045)	0.251	(0.044)	0.214	(0.046)	0.179	(0.048)	0.195	(0.040)
6	Yamagata	0.168	(0.040)	0.157	(0.052)	0.126	(0.041)	0.099	(0.036)	0.120	(0.027)
7	Fukushima	0.130	(0.050)	0.120	(0.044)	0.137	(0.061)	0.137	(0.054)	0.114	(0.047)
8	Ibaraki	0.094	(0.049)	0.071	(0.068)	0.086	(0.035)	0.074	(0.057)	0.098	(0.036)
9	Tochigi	0.117	(0.050)	0.103	(0.049)	0.104	(0.040)	0.092	(0.045)	0.118	(0.031)
10	Gunma	0.047	(0.046)	0.032	(0.035)	0.047	(0.073)	0.036	(0.063)	0.050	(0.036)
11	Saitama	0.009	(0.060)	-0.001	(0.030)	-0.001	(0.050)	0.003	(0.043)	-0.003	(0.029)
12	Chiba	0.016	(0.041)	0.010	(0.045)	0.031	(0.056)	0.028	(0.054)	0.013	(0.023)
13	Tokyo	-0.003	(0.040)	-0.008	(0.038)	-0.019	(0.044)	-0.008	(0.040)	-0.017	(0.036)
14	Kanagawa	-0.050	(0.036)	-0.035	(0.039)	-0.033	(0.043)	-0.023	(0.045)	-0.037	(0.048)
15	Niigata	0.066	(0.042)	0.042	(0.046)	0.030	(0.048)	0.039	(0.042)	0.043	(0.045)
16	Toyama	0.091	(0.048)	0.084	(0.036)	0.081	(0.040)	0.066	(0.047)	0.056	(0.041)
17	Ishikawa	0.041	(0.059)	0.026	(0.065)	0.036	(0.053)	0.026	(0.039)	0.049	(0.039)
18	Fukui	0.001	(0.032)	0.013	(0.051)	0.016	(0.034)	-0.013	(0.038)	0.008	(0.033)
19	Yamanashi	-0.022	(0.062)	-0.024	(0.054)	-0.021	(0.055)	-0.060	(0.054)	-0.009	(0.039)
20	Nagano	-0.092	(0.038)	-0.086	(0.049)	-0.095	(0.035)	-0.091	(0.049)	-0.041	(0.038)
21	Gifu	-0.068	(0.049)	-0.070	(0.038)	-0.083	(0.050)	-0.055	(0.047)	-0.065	(0.033)
22	Shizuoka	-0.093	(0.049)	-0.062	(0.055)	-0.067	(0.043)	-0.076	(0.041)	-0.055	(0.052)
23	Aich	-0.095	(0.036)	-0.091	(0.036)	-0.095	(0.058)	-0.103	(0.037)	-0.083	(0.028)
24	Mie	-0.034	(0.054)	-0.038	(0.023)	-0.046	(0.047)	-0.013	(0.051)	-0.063	(0.035)
25	Shiga	-0.063	(0.044)	-0.084	(0.060)	-0.088	(0.038)	-0.088	(0.037)	-0.077	(0.029)
26	Kyoto	-0.092	(0.038)	-0.079	(0.039)	-0.062	(0.049)	-0.063	(0.053)	-0.086	(0.030)
27	Osaka	-0.039	(0.047)	-0.050	(0.038)	-0.033	(0.049)	-0.029	(0.059)	-0.040	(0.039)
28	Hyogo	-0.064	(0.038)	-0.057	(0.040)	-0.095	(0.059)	-0.106	(0.041)	-0.077	(0.025)
29	Nara	-0.028	(0.058)	-0.045	(0.065)	-0.027	(0.062)	-0.048	(0.044)	-0.030	(0.049)
30	Wakayama	-0.015	(0.042)	0.016	(0.048)	0.004	(0.045)	0.003	(0.054)	0.009	(0.066)
31	Tottori	-0.011	(0.045)	-0.003	(0.049)	-0.015	(0.062)	-0.025	(0.053)	-0.022	(0.043)
32	Shimane	-0.070	(0.080)	-0.075	(0.056)	-0.077	(0.064)	-0.088	(0.039)	-0.082	(0.032)
33	Okayama	-0.062	(0.036)	-0.062	(0.048)	-0.074	(0.041)	-0.058	(0.063)	-0.050	(0.050)
34	Hiroshima	-0.040	(0.054)	-0.067	(0.046)	-0.040	(0.045)	-0.029	(0.057)	-0.028	(0.029)
35	Yamaguchi	-0.079	(0.047)	-0.102	(0.056)	-0.100	(0.046)	-0.075	(0.060)	-0.100	(0.034)
36	Tokushima	0.091	(0.058)	0.091	(0.047)	0.118	(0.067)	0.109	(0.046)	0.109	(0.031)
37	Kagawa	-0.024	(0.062)	-0.014	(0.060)	0.014	(0.034)	-0.004	(0.062)	0.045	(0.029)
38	Ehime	-0.058	(0.031)	-0.026	(0.042)	-0.046	(0.044)	-0.058	(0.056)	0.005	(0.033)
39	Kochi	-0.061	(0.044)	-0.015	(0.069)	-0.024	(0.056)	0.000	(0.046)	0.023	(0.053)
40	Fukuoka	-0.028	(0.053)	-0.033	(0.057)	-0.026	(0.054)	-0.027	(0.055)	-0.043	(0.035)
41	Saga	-0.029	(0.042)	-0.008	(0.051)	0.000	(0.043)	-0.001	(0.079)	0.003	(0.047)
42	Nagasaki	-0.043	(0.050)	-0.041	(0.054)	-0.065	(0.047)	-0.064	(0.038)	-0.048	(0.053)
43	Kumamoto	0.033	(0.058)	0.024	(0.053)	0.002	(0.066)	0.015	(0.049)	0.003	(0.051)
44	Oita	0.006	(0.047)	0.011	(0.084)	0.016	(0.050)	-0.009	(0.059)	0.002	(0.045)
45	Miyazaki	0.006	(0.065)	-0.029	(0.054)	0.003	(0.058)	0.017	(0.062)	0.000	(0.057)
46	Kagoshima	-0.059	(0.066)	-0.065	(0.045)	-0.045	(0.050)	-0.048	(0.067)	-0.024	(0.045)
47	Okinawa	-0.016	(0.058)	0.013	(0.062)	0.071	(0.060)	0.032	(0.035)	0.020	(0.045)

Table S2.
Standardized 2eight in each prefecture (averaged over a 13-year period from 1996 to 2008, 13- to 17-year-old: Females)

		Female									
No.	Prefecture	13	(SE)	14	(SE)	15	(SE)	16	(SE)	17	(SE)
1	Hokkaido	0.104	(0.035)	0.068	(0.046)	0.058	(0.054)	0.065	(0.069)	0.066	(0.048)
2	Aomori	0.252	(0.057)	0.231	(0.030)	0.200	(0.058)	0.186	(0.075)	0.168	(0.055)
3	Iwate	0.137	(0.043)	0.135	(0.048)	0.157	(0.040)	0.169	(0.049)	0.197	(0.052)
4	Miyagi	0.139	(0.035)	0.139	(0.040)	0.147	(0.079)	0.139	(0.067)	0.154	(0.064)
5	Akita	0.192	(0.044)	0.171	(0.032)	0.180	(0.054)	0.154	(0.057)	0.156	(0.064)
6	Yamagata	0.128	(0.029)	0.142	(0.032)	0.186	(0.081)	0.186	(0.052)	0.189	(0.063)
7	Fukushima	0.108	(0.043)	0.123	(0.056)	0.148	(0.058)	0.179	(0.057)	0.128	(0.055)
8	Ibaraki	0.059	(0.036)	0.090	(0.040)	0.103	(0.047)	0.074	(0.043)	0.082	(0.050)
9	Tochigi	0.104	(0.031)	0.121	(0.045)	0.119	(0.047)	0.111	(0.081)	0.103	(0.072)
10	Gunma	0.026	(0.038)	0.052	(0.032)	0.060	(0.077)	0.066	(0.085)	0.074	(0.059)
11	Saitama	0.011	(0.043)	0.025	(0.036)	0.047	(0.048)	0.044	(0.056)	0.004	(0.055)
12	Chiba	0.035	(0.032)	0.028	(0.038)	0.052	(0.046)	0.025	(0.057)	0.000	(0.044)
13	Tokyo	0.001	(0.033)	-0.037	(0.036)	-0.040	(0.053)	-0.053	(0.034)	-0.041	(0.065)
14	Kanagawa	-0.030	(0.034)	-0.045	(0.035)	-0.072	(0.037)	-0.068	(0.046)	-0.052	(0.058)
15	Niigata	0.024	(0.048)	0.048	(0.026)	0.027	(0.054)	0.056	(0.032)	0.066	(0.054)
16	Toyama	0.065	(0.028)	0.058	(0.040)	0.092	(0.059)	0.095	(0.049)	0.069	(0.056)
17	Ishikawa	0.029	(0.031)	0.027	(0.045)	0.046	(0.039)	0.081	(0.056)	0.048	(0.038)
18	Fukui	0.027	(0.027)	0.044	(0.029)	0.059	(0.078)	0.062	(0.048)	0.059	(0.043)
19	Yamanashi	-0.004	(0.036)	0.000	(0.046)	0.019	(0.039)	-0.013	(0.052)	0.002	(0.054)
20	Nagano	-0.023	(0.046)	0.006	(0.027)	0.001	(0.040)	-0.011	(0.053)	-0.008	(0.045)
21	Gifu	-0.067	(0.035)	-0.052	(0.050)	-0.099	(0.043)	-0.080	(0.056)	-0.121	(0.047)
22	Shizuoka	-0.056	(0.042)	-0.030	(0.036)	-0.027	(0.062)	-0.074	(0.054)	-0.039	(0.043)
23	Aich	-0.067	(0.026)	-0.054	(0.021)	-0.093	(0.043)	-0.077	(0.053)	-0.082	(0.041)
24	Mie	-0.053	(0.029)	-0.086	(0.042)	-0.054	(0.059)	-0.070	(0.062)	-0.045	(0.061)
25	Shiga	-0.057	(0.026)	-0.038	(0.026)	-0.009	(0.034)	0.040	(0.043)	0.046	(0.042)
26	Kyoto	-0.068	(0.044)	-0.064	(0.029)	-0.040	(0.060)	-0.028	(0.038)	-0.029	(0.056)
27	Osaka	-0.002	(0.024)	-0.026	(0.051)	-0.024	(0.038)	-0.017	(0.038)	-0.009	(0.053)
28	Hyogo	-0.063	(0.035)	-0.042	(0.037)	-0.007	(0.052)	-0.040	(0.046)	-0.017	(0.052)
29	Nara	-0.021	(0.031)	-0.037	(0.040)	0.013	(0.049)	-0.017	(0.067)	0.007	(0.064)
30	Wakayama	-0.004	(0.039)	-0.018	(0.036)	0.012	(0.058)	0.020	(0.064)	0.006	(0.081)
31	Tottori	-0.006	(0.046)	0.000	(0.042)	-0.026	(0.046)	-0.026	(0.065)	-0.015	(0.035)
32	Shimane	-0.087	(0.038)	-0.075	(0.034)	-0.069	(0.052)	-0.065	(0.061)	-0.054	(0.055)
33	Okayama	-0.058	(0.039)	-0.047	(0.029)	-0.034	(0.065)	-0.046	(0.041)	-0.041	(0.033)
34	Hiroshima	-0.047	(0.035)	-0.060	(0.043)	-0.071	(0.077)	-0.062	(0.058)	-0.039	(0.056)
35	Yamaguchi	-0.121	(0.028)	-0.100	(0.040)	-0.101	(0.053)	-0.112	(0.076)	-0.110	(0.057)
36	Tokushima	0.110	(0.051)	0.096	(0.033)	0.045	(0.074)	0.069	(0.051)	0.046	(0.055)
37	Kagawa	0.031	(0.029)	0.054	(0.045)	0.017	(0.070)	0.008	(0.049)	-0.004	(0.048)
38	Ehime	-0.020	(0.041)	-0.007	(0.054)	-0.021	(0.040)	-0.016	(0.041)	0.004	(0.062)
39	Kochi	-0.015	(0.045)	-0.063	(0.052)	-0.058	(0.040)	-0.041	(0.039)	-0.065	(0.061)
40	Fukuoka	-0.052	(0.032)	-0.074	(0.027)	-0.063	(0.066)	-0.032	(0.063)	-0.060	(0.049)
41	Saga	-0.003	(0.041)	-0.017	(0.031)	0.002	(0.047)	0.018	(0.065)	0.013	(0.046)
42	Nagasaki	-0.018	(0.056)	-0.013	(0.023)	-0.027	(0.073)	-0.005	(0.064)	-0.019	(0.057)
43	Kumamoto	-0.022	(0.047)	-0.023	(0.051)	-0.021	(0.054)	-0.013	(0.063)	-0.011	(0.051)
44	Oita	-0.005	(0.062)	-0.012	(0.052)	0.014	(0.049)	-0.011	(0.055)	0.008	(0.061)
45	Miyazaki	-0.022	(0.043)	-0.026	(0.044)	-0.002	(0.065)	-0.012	(0.061)	-0.040	(0.066)
46	Kagoshima	-0.033	(0.046)	-0.022	(0.042)	-0.034	(0.035)	-0.002	(0.033)	0.010	(0.051)
47	Okinawa	-0.051	(0.048)	-0.105	(0.051)	-0.168	(0.047)	-0.236	(0.068)	-0.238	(0.066)
