# Supplementary material for: Geographical Distribution of Adolescent Body Height with Respect to Effective Day Length in Japan: An Ecological Analysis
Source: PLoS One. 2012 Dec 5;7(12):e50994. doi: 10.1371/journal.pone.0050994 (PMC3515496; doi:10.1371/journal.pone.0050994)
Supplement: Table S3 — Population-weighted averaged climatic values derived from mesh climatic data in each prefecture. (PDF) [file pone.0050994.s003.pdf]

Table S3.
Population-weighted average climatic value in each prefecture

No.	Prefecture	TEMP	SOLA	SUN	1000	2000	3000	4000	5000	10000
1	Hokkaido	7.1	12.1	1783	11.62	11.25	10.84	10.47	10.10	8.21
2	Aomori	9.7	12.2	1761	11.63	11.24	10.86	10.49	10.11	8.23
3	Iwate	9.8	12.1	1727	11.62	11.25	10.89	10.52	10.14	8.30
4	Miyagi	11.5	12.3	1859	11.65	11.30	10.95	10.60	10.24	8.47
5	Akita	10.5	11.8	1584	11.61	11.19	10.80	10.42	10.03	8.05
6	Yamagata	11.0	12.0	1601	11.62	11.24	10.85	10.48	10.09	8.19
7	Fukushima	11.6	12.8	1836	11.64	11.32	10.97	10.63	10.29	8.56
8	Ibaraki	13.5	12.8	1884	11.65	11.32	10.98	10.64	10.30	8.64
9	Tochigi	12.9	12.6	1954	11.65	11.31	10.96	10.63	10.31	8.60
10	Gunma	13.7	12.7	2011	11.65	11.33	10.97	10.66	10.31	8.63
11	Saitama	14.4	12.3	1893	11.64	11.30	10.95	10.60	10.26	8.53
12	Chiba	14.7	12.3	1852	11.64	11.29	10.95	10.59	10.24	8.48
13	Tokyo	15.0	12.1	1858	11.64	11.30	10.93	10.57	10.21	8.44
14	Kanagawa	15.1	12.3	1883	11.64	11.29	10.94	10.59	10.24	8.49
15	Niigata	12.6	11.7	1570	11.60	11.19	10.82	10.42	10.03	8.06
16	Toyama	13.3	12.0	1582	11.62	11.25	10.85	10.49	10.11	8.21
17	Ishikawa	13.8	12.3	1654	11.63	11.25	10.89	10.49	10.12	8.27
18	Fukui	13.9	12.1	1589	11.62	11.25	10.88	10.51	10.14	8.28
19	Yamanashi	12.8	13.5	2044	11.69	11.35	11.06	10.74	10.43	8.84
20	Nagano	10.7	13.6	1956	11.69	11.36	11.05	10.73	10.42	8.82
21	Gifu	14.0	13.1	1950	11.67	11.34	11.00	10.68	10.34	8.71
22	Shizuoka	15.5	13.3	1999	11.69	11.36	11.04	10.72	10.39	8.79
23	Aich	15.2	13.3	2067	11.69	11.35	11.04	10.72	10.39	8.79
24	Mie	14.8	12.8	1960	11.66	11.33	10.99	10.66	10.32	8.64
25	Shiga	13.9	12.4	1774	11.62	11.28	10.93	10.57	10.21	8.44
26	Kyoto	14.7	12.1	1743	11.63	11.28	10.90	10.56	10.19	8.38
27	Osaka	15.7	12.5	1952	11.64	11.29	10.95	10.61	10.27	8.50
28	Hyogo	14.9	12.6	1921	11.64	11.29	10.97	10.62	10.27	8.54
29	Nara	14.4	12.5	1830	11.64	11.29	10.94	10.59	10.24	8.46
30	Wakayama	15.6	13.0	1965	11.65	11.32	10.99	10.66	10.32	8.66
31	Tottori	14.0	12.4	1694	11.64	11.28	10.90	10.55	10.18	8.37
32	Shimane	14.0	12.7	1728	11.64	11.29	10.91	10.57	10.22	8.44
33	Okayama	14.5	13.2	1970	11.68	11.34	11.01	10.68	10.35	8.70
34	Hiroshima	14.6	13.3	1932	11.69	11.35	11.02	10.71	10.37	8.77
35	Yamaguchi	15.0	13.5	1977	11.68	11.34	11.04	10.70	10.39	8.77
36	Tokushima	15.4	13.1	1970	11.67	11.32	11.00	10.67	10.34	8.70
37	Kagawa	15.4	13.4	2046	11.69	11.35	11.03	10.70	10.39	8.76
38	Ehime	15.5	13.5	1911	11.68	11.34	11.04	10.72	10.39	8.78
39	Kochi	15.9	14.0	2104	11.70	11.39	11.10	10.80	10.50	9.03
40	Fukuoka	15.7	12.8	1835	11.66	11.31	10.99	10.63	10.29	8.59
41	Saga	15.4	13.1	1876	11.67	11.34	11.01	10.68	10.34	8.69
42	Nagasaki	15.9	13.1	1874	11.67	11.34	11.00	10.68	10.34	8.69
43	Kumamoto	15.7	13.2	1926	11.68	11.34	11.02	10.70	10.37	8.74
44	Oita	15.1	13.0	1908	11.67	11.33	11.02	10.68	10.33	8.69
45	Miyazaki	16.4	13.8	2034	11.70	11.40	11.08	10.77	10.47	8.98
46	Kagoshima	17.3	13.4	1880	11.69	11.36	11.04	10.74	10.41	8.84
47	Okinawa	22.3	13.8	1775	11.69	11.39	11.09	10.76	10.44	8.90

TMP: Annual mean temperature (°C)
SOLA: Annualm ean solar radiation (MJ/m2/day)
SUN: Annual total sunshine duration (h/year)
1000-10000: Annual mean effective day length at 1000-10000 lx (h/day)
